# The autism-associated chromatin modifier CHD8 regulates other autism risk genes during human neurodevelopment

**DOI:** 10.1038/ncomms7404

**Published:** 2015-03-10

**Authors:** Justin Cotney, Rebecca A. Muhle, Stephan J. Sanders, Li Liu, A. Jeremy Willsey, Wei Niu, Wenzhong Liu, Lambertus Klei, Jing Lei, Jun Yin, Steven K. Reilly, Andrew T. Tebbenkamp, Candace Bichsel, Mihovil Pletikos, Nenad Sestan, Kathryn Roeder, Matthew W. State, Bernie Devlin, James P. Noonan

**Affiliations:** 1 Department of Genetics, Yale School of Medicine, 333 Cedar Street, New Haven, Connecticut 06510, USA; 2 Kavli Institute for Neuroscience, Yale School of Medicine, PO Box 208001, New Haven, Connecticut 06520, USA; 3 Child Study Center, Yale School of Medicine, 230S. Frontage Road, New Haven, Connecticut 06519, USA; 4 Department of Psychiatry, University of California, 401 Parnassus Avenue, San Francisco, California 94143, USA; 5 Department of Statistics, Carnegie Mellon University, Baker Hall 228B, Pittsburgh, Pennsylvania 15213, USA; 6 Department of Psychiatry, University of Pittsburgh School of Medicine, 3811 O'Hara Street, Pittsburgh, Pennsylvania 15213, USA; 7 Department of Neurobiology, Yale School of Medicine, PO Box 208001, New Haven, Connecticut 06510, USA; 8 Ray and Stephanie Lane Center for Computational Biology, Carnegie Mellon University, 7401 Gates-Hillman Center, 5000 Forbes Avenue, Pittsburgh, Pennsylvania 15213, USA

## Abstract

Recent studies implicate chromatin modifiers in autism spectrum disorder (ASD) through the identification of recurrent *de novo* loss of function mutations in affected individuals. ASD risk genes are co-expressed in human midfetal cortex, suggesting that ASD risk genes converge in specific regulatory networks during neurodevelopment. To elucidate such networks, we identify genes targeted by CHD8, a chromodomain helicase strongly associated with ASD, in human midfetal brain, human neural stem cells (hNSCs) and embryonic mouse cortex. CHD8 targets are strongly enriched for other ASD risk genes in both human and mouse neurodevelopment, and converge in ASD-associated co-expression networks in human midfetal cortex. CHD8 knockdown in hNSCs results in dysregulation of ASD risk genes directly targeted by CHD8. Integration of CHD8-binding data into ASD risk models improves detection of risk genes. These results suggest loss of CHD8 contributes to ASD by perturbing an ancient gene regulatory network during human brain development.

## Abstract (web summary)

Autism genes converge in midfetal cortical co-expression networks, and chromatin regulators such as CHD8 are increasingly associated with autism spectrum disorder (ASD). Here the authors map CHD8 targets in developing brain, and find that CHD8 directly regulates other ASD risk genes during human neurodevelopment.

## Graphical abstract

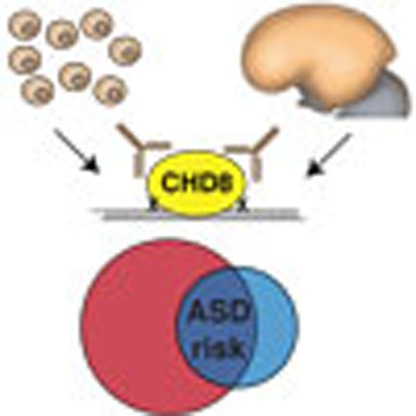

The molecular and cellular pathology underlying the development of autism spectrum disorder (ASD) remains poorly understood. The genetic heterogeneity of ASD has made it challenging to identify specific genes associated with the disorder, which has thus hindered efforts to dissect disease mechanisms^1^,^2^,^3^,^4^. However, two recent developments have sparked rapid progress in ASD gene discovery. First, it is now appreciated that *de novo* mutations contribute to ASD and often carry large effects^5^,^6^,^7^,^8^. Second, the advent of next-generation sequencing technologies has enabled hypothesis-naïve whole-exome surveys of large ASD cohorts to identify genes with *de novo*, ASD-associated damaging mutations^9^,^10^,^11^,^12^. This approach allows the level of ASD risk to be assessed for all genes using uniform statistical and genetic criteria, providing a quantitative definition of an ASD risk gene independent of prior hypotheses regarding gene functions or disease processes.

Initial sequencing studies established that genes with multiple *de novo* loss of function mutations among unrelated persons with ASD are highly likely to confer risk for the disorder. To date, nine such high-confidence^13^ ASD risk genes have been identified: *ANK2, CHD8, CUL3, DYRK1A, GRIN2B, KATNAL2, POGZ, SCN2A* and *TBR1*. These genes encode proteins with a variety of functions, including chromatin modification and transcriptional regulation^14^, suggesting molecular mechanisms perturbed in ASD. Of these genes, *CHD8* has the largest number of loss of function mutations in individuals with ASD, and therefore the strongest association with ASD risk. Eleven independent *de novo* loss of function mutations in *CHD8* have been identified in unrelated individuals with ASD^9^,^11^,^15^,^16^.

*CHD8* encodes an ATP-dependent chromatin remodeller that binds to trimethylated histone H3 lysine 4, a post-translational histone modification present at active promoters^17^,^18^,^19^. CHD8 has also been shown to bind promoters of E2 adenovirus promoter binding factor-target genes and is required for their expression during the G1/S transition of the cell cycle^20^. Other studies suggest CHD8 may repress Wnt/β-catenin target genes and p53-dependent apoptosis^17^,^21^. These findings, coupled with the strong genetic evidence described above, suggest that loss of CHD8 function contributes to ASD pathology by disrupting the expression of genes regulated by CHD8.

Recent studies also suggest that ASD risk genes converge in gene co-expression networks in the developing human brain, providing further support for a gene regulatory contribution to ASD aetiology^13^,^22^. Willsey *et al*.^13^ used a rich data set of gene expression throughout human brain development to identify networks of genes that were co-expressed with the nine known high-confidence ASD risk genes at specific brain regions and points in time. To define a larger set of potential ASD risk genes, Willsey *et al*. identified 122 genes that had a *de novo* loss of function in a single individual with ASD, but not in matched controls. These potential ASD risk genes show the most significant co-expression with high-confidence ASD risk genes in midfetal prefrontal and primary motor-somatosensory cortex (PFC-MSC). A parallel study also supported the convergence of ASD risk genes in co-expression networks at this developmental time point and location^22^. These findings suggest ASD risk genes are co-regulated, and may thus converge in regulatory networks associated with ASD. Owing to its chromatin remodelling activity, its association with other transcriptional regulators, and its increased expression during human midfetal development^15^, CHD8 is a prime candidate for contributing to the organization of such networks by regulating other ASD risk genes.

This study investigates the role of CHD8 in regulating other ASD risk genes in human neurodevelopment. Although a recent study suggested that CHD8 targets ASD risk genes in human neural progenitors derived from induced pluripotent stem cells^23^, CHD8 binding has not been examined in developing human brain at the stages most relevant for ASD pathology. The extent to which CHD8 may directly regulate other ASD risk genes *in vivo* remains unknown. We therefore posed three questions regarding CHD8 function and its relevance to autism. First, are ASD risk genes overrepresented among genes targeted by CHD8 in the developing brain? Second, are CHD8 targets overrepresented in ASD-associated co-expression networks in midfetal human brain? Third, does loss of CHD8 result in dysregulation of ASD risk genes that are targeted by CHD8? To address these questions we utilized two resources: representative human neurodevelopmental tissues in which CHD8 gene targets can be mapped or CHD8 expression perturbed; and uniformly defined sets of ASD risk genes to query sets of CHD8 gene targets for autism risk. To identify CHD8 gene targets, we used chromatin immunoprecipitation followed by high-throughput sequencing (ChIP-seq) to map CHD8-binding sites in human midfetal brain, human neural stem cells (hNSCs) and mouse embryonic cortex (for experimental schematic see Supplementary Fig. 1). The hNSC model system provides the means to directly perturb CHD8 expression and evaluate consequent effects on CHD8 target genes. To assess whether ASD risk genes are overrepresented among the CHD8 targets we identified, we used two sets of ASD risk genes previously described in the literature. The first is the list of potential ASD risk genes described by Willsey *et al*. The second list, described by Liu *et al*., incorporates *de novo* and transmitted mutations from ASD exome sequencing, genetic data from ASD case–control studies and gene co-expression in midfetal human brain into a statistical model that improves discrimination of ASD risk genes^24^. The advantage of these lists is that they were ascertained via genome-wide hypothesis-naïve approaches for defining ASD risk using consistent statistical criteria.

We identify a highly conserved set of CHD8 targets in the developing mammalian brain that is strongly enriched in ASD risk genes. CHD8 gene targets are overrepresented in the ASD-associated co-expression network identified in human midfetal brain^13^, supporting the hypothesis that CHD8 is a key regulator of genes in this network. After downregulation of CHD8 expression in hNSCs, ASD risk genes bound by CHD8 in multiple neurodevelopmental contexts are significantly dysregulated by CHD8 loss. Finally, integrating CHD8 binding with genetic and co-expression data into the predictive model described in Liu *et al*. improves identification of genes harbouring risk for ASD (Supplementary Fig. 1). Taken together, these multiple lines of evidence support CHD8 as a direct regulator of other ASD risk genes during human brain development.

## Results

### CHD8 target sites identified during human neurodevelopment

**Figure 1:**
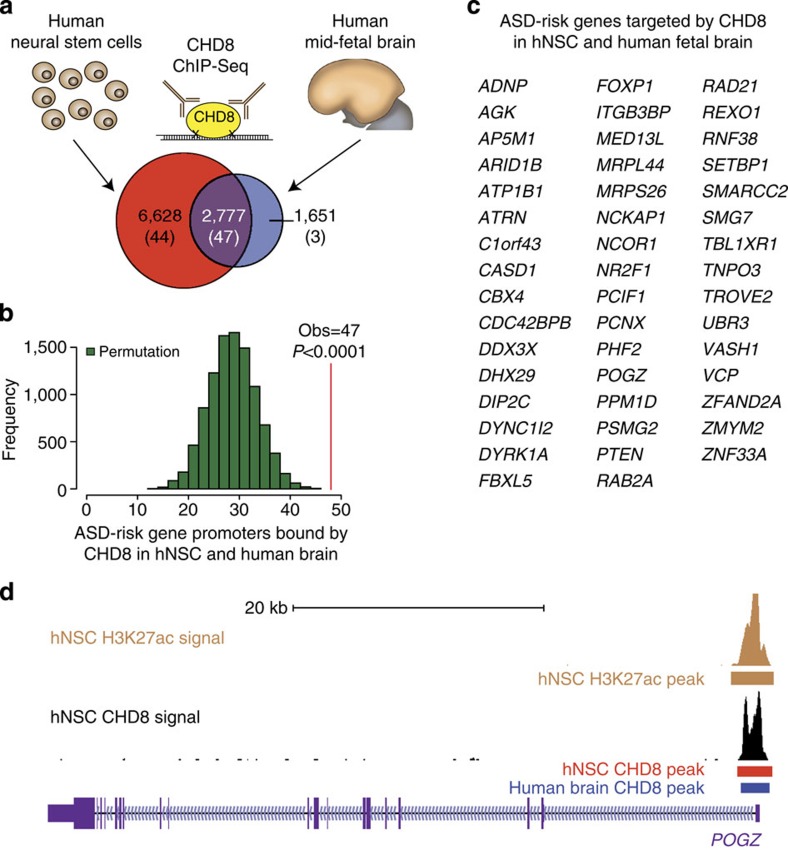
CHD8 targets in neurodevelopment are enriched for ASD risk genes. (**a**) Reproducible CHD8-binding sites identified in two biological replicates of hNSC and midfetal human brain. The number of reproducible sites in each tissue and the subset identified in both tissues are indicated in each section of the Venn diagram. The number of ASD risk genes from Liu *et al*. bound by CHD8 in each subset is noted in parentheses. (**b**) Histogram showing the results of ASD risk gene label permutations (*n*=10,000, green bars) assessing enrichment of ASD risk genes reported by Liu *et al*. within targets of CHD8 shared in hNSCs and midfetal human brain. The observed number of ASD risk genes identified is indicated by a vertical red line. (**c**) List of ASD risk genes identified by Liu *et al*. with shared CHD8 binding between hNSCs and midfetal human brain (*n*=47). (**d**) Representative ChIP-Seq signal tracks for H3K27ac and CHD8 from hNSCs at the high-confidence ASD gene *POGZ*. CHD8 peak calls from hNSCs and midfetal human brain are indicated by horizontal bars. CHD8 binding is coincident with strong H3K27ac signal surrounding the transcription start site in hNSCs.

Using ChIP-seq with an antibody targeting an N-terminal epitope of CHD8 (Supplementary Fig. 2a,b), we identified CHD8-binding sites in H9-derived human NSCs and human midfetal brain at 16–19 post conception weeks (PCWs). As described above, potential ASD risk genes converge in co-expression networks at this developmental stage^13^,^22^. In hNSC, CHD8 binding was reproducibly identified at 9,414 sites across the human genome and was enriched at promoters versus more distal genomic sites (Fig. 1a, Supplementary Fig. 3a,b and Supplementary Data 1). We identified 4,428 reproducible binding sites in human midfetal brain, most of which also overlap with promoters (Fig. 1a, Supplementary Fig. 3a,b and Supplementary Data 1). Many of the CHD8-binding sites in human midfetal brain were shared with hNSCs, identifying a set of genes that are targeted by CHD8 in both neurodevelopmental contexts (Fig. 1a).

To gain an initial view of the potential regulatory role of CHD8 at its target genes, we considered the co-occurrence of CHD8 binding with histone modifications associated with either active or repressed chromatin. Using data generated in our own lab as well as publicly available data sets for hNSCs^25^, we found that 99% (8056) of promoters bound by CHD8 in hNSCs were enriched for the active chromatin marks H3K4me3 or H3K27ac^26^ (Supplementary Data 1 and Supplementary Fig. 3c). Consistent with this finding, the level of CHD8 binding at promoters was positively correlated with the level of gene expression (Supplementary Fig. 3d). We observed little enrichment for H3K9me3 and a negative correlation with H3K27me3, both marks associated with repressed chromatin states^27^, at promoters bound by CHD8 in hNSCs (Supplementary Fig. 3e). Although distal CHD8-binding sites represent a smaller fraction of the data, 90% (1,028) of distal sites were marked with active histone modifications, suggesting they are CHD8-bound enhancers (Supplementary Data 1 and Supplementary Fig. 3f). These data indicate that CHD8 is found primarily at the promoters of actively transcribed genes in neurodevelopmental tissues.

A detailed investigation of the mechanisms of CHD8-dependent gene regulation is beyond the scope of this study. However, CHD8 has been shown to interact directly with E2F^20^ and CTCF^28^ in non-neuronal biological contexts. To evaluate whether CHD8 may cooperate with these factors in a neurodevelopmental context, we searched for enriched transcription factor motifs surrounding CHD8-binding sites overlapping promoters in both hNSC and human midfetal brain. As expected, motifs for CTCF and E2F were significantly enriched. In addition, we found that binding sites of the transcription factor YY1 and Sp/Kruppel-like family of transcription factors were also very strongly enriched, suggesting these factors may play a role in CHD8-mediated gene regulation in the brain (Supplementary Data 2).

### Human CHD8 targets are enriched for ASD risk genes

Having established that CHD8 has the potential to regulate gene expression in human neurodevelopment, we next sought to determine whether CHD8 targets were enriched for genes associated with ASD risk. We independently determined the overlap between CHD8 targets in each tissue and the lists of ASD risk genes identified by Willsey *et al*. and Liu *et al*. Surprisingly, we found the greatest apparent excess of ASD risk genes from each list among CHD8 targets bound in both hNSC and human midfetal brain (Fig. 1a and Supplementary Fig. 4a). We then performed permutation tests to determine whether ASD risk genes from each list were significantly enriched. In each iteration, we permuted ASD risk genes by randomly selecting the same number of genes from the genome while controlling for gene size, GC content and promoter activation in hNSCs; we then counted the number of randomly selected genes whose promoters are bound by CHD8. Of 127 analysed ASD risk genes from Liu *et al*., 47 are targeted by CHD8 in both human tissues (permutation test *P* value<0.0001, Fig. 1b,c, Supplementary Fig. 5 and Supplementary Data 3). The 116 analysed ASD risk genes from Willsey *et al*. were also significantly enriched among these CHD8 targets (46 targeted risk genes, permutation test *P* value<0.0001, Supplementary Figs 4b and 5, and Supplementary Data 3). In contrast, CHD8 targets specific to hNSCs were not enriched for ASD risk genes from either list (Supplementary Fig. 5, permutation test *P*=0.9911). This finding highlights the power of *in vivo* CHD8-binding data for understanding the role of CHD8 in ASD. We also permuted CHD8-binding events across gene promoters and obtained similar results, reinforcing the robustness of the enrichments we detected (Supplementary Fig. 5 and Supplementary Data 3). Together, these results suggest that CHD8 targets a subset of ASD risk genes in the developing human brain.

**Figure 2:**
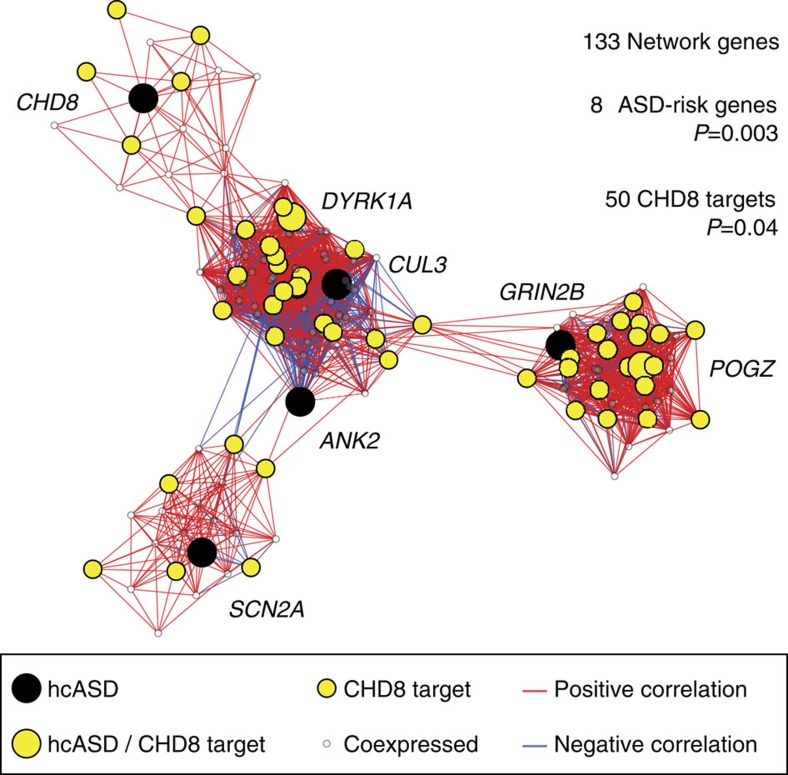
CHD8 targets are enriched in an ASD-associated co-expression network. A gene co-expression network spanning 10–19 post conception weeks (defined as Periods 3–5 in Willsey *et al*.) was constructed as described^13^,^46^, except the set of input genes was further restricted to only include genes exhibiting H3K27ac and/or H3K4me3 promoter marking in hNSCs to match the observed characteristics of CHD8 targets. The resulting network was tested for enrichment of potential ASD genes identified by Willsey *et al*., and genes with CHD8-binding sites in their promoters. The 20 genes best correlated with each high-confidence ASD gene (‘hcASD gene’) were included in the network provided the correlation value was *R*≥0.7. The hcASD seed genes are shown as large circles; CHD8 targets are in yellow; and the top 20 genes that are not CHD8 targets are small white circles. The lines (edges) reflect co-expression correlations: positive correlations are in red and negative correlations are in blue.

We next sought to determine whether CHD8 targets in human neurodevelopment were enriched in ASD-associated co-expression networks previously identified in human midfetal prefrontal and primary motor-somatosensory cortex^13^,^22^. To ensure that any observed enrichment was not driven by overrepresentation of active promoters within the co-expression network, we reconstructed the 10–19 PCW network described in Willsey *et al*. using only genes with active promoters in hNSC. CHD8 targets were significantly enriched in the resulting network, as were ASD risk genes identified by Willsey *et al*. (Fig. 2). Similar enrichments were obtained for the ASD-associated 13–24 PCW network identified in the previous study (Supplementary Fig. 6). These findings support a regulatory role for CHD8 in co-expression networks during human brain development that are enriched in genes potentially associated with ASD. Loss of CHD8 may disrupt these networks and thereby contribute to ASD aetiology.

### Conservation of CHD8 binding in neurodevelopment

**Figure 3:**
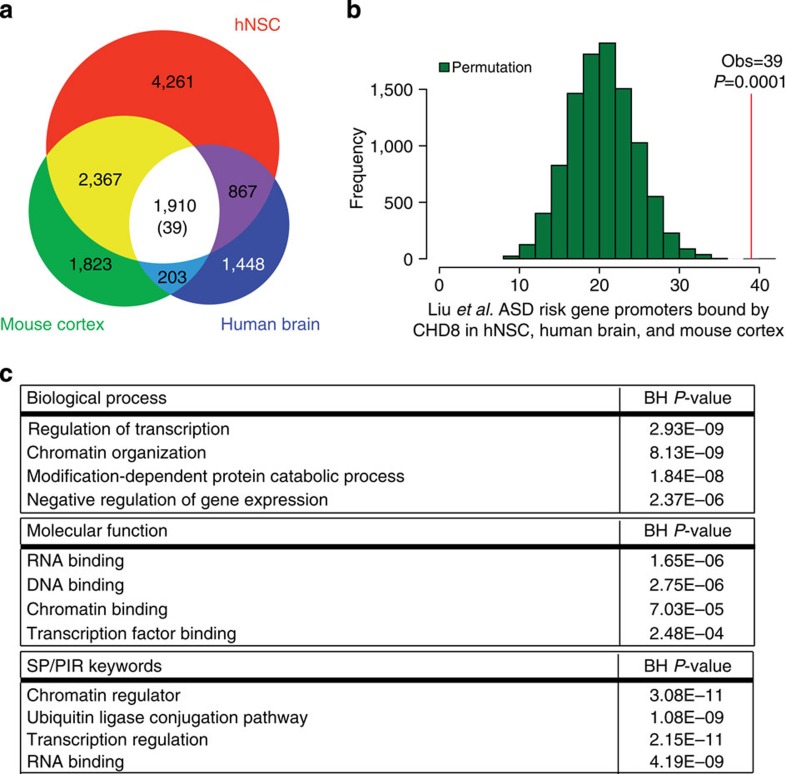
Conserved CHD8-binding sites are enriched for ASD risk genes. (**a**) Reproducible CHD8-binding sites identified in two biological replicates of hNSC, midfetal human brain and embryonic day 17.5 mouse cortex. The number of reproducible sites in each tissue and the subset identified in both tissues are indicated in each section of the Venn diagram. The number of ASD risk genes from Liu *et al*. bound by CHD8 in each subset is noted in parentheses. (**b**) Histogram showing the results of ASD risk gene label permutations (*n*=10,000, green bars) assessing enrichment of ASD risk genes reported by Liu *et al*. within conserved CHD8 target genes. (**c**) Selected gene ontology categories reported by DAVID^36^ as enriched in the set of genes bound by CHD8 in hNSC, midfetal human brain and mouse cortex. *P* values were corrected for multiple testing using the Benjamini–Hochberg method.

To determine whether CHD8 targeting of ASD risk genes was a conserved feature of mammalian brain development, we also mapped CHD8 targets in the mouse embryonic day 17.5 cortex. We identified 1,910 CHD8-binding sites that are shared among human midfetal brain, mouse cortex and hNSCs (Fig. 3a). Using the same permutation approach described above, we found that ASD risk genes identified by Willsey *et al*. or Liu *et al*. were significantly enriched in this conserved set of CHD8 targets (39 from Liu *et al*., 37 from Willsey *et al*., permutation test *P*<0.0001 for each list, Fig. 3b, Supplementary Fig. 5 and Supplementary Data 3). These findings support a highly conserved role for CHD8 in regulating other ASD risk genes during mammalian neurodevelopment.

To elucidate potential biological functions of genes regulated by CHD8, we carried out gene ontology enrichment analyses on conserved CHD8 targets. These target genes were strongly enriched for functions related to transcriptional regulation and chromatin modification (Fig. 3c and Supplementary Data 4). Notably, many of the ASD risk genes targeted by CHD8 include chromatin modifiers and transcription factors (Fig. 1c and Supplementary Fig. 4b). We observed similar enrichments for all genes targeted by CHD8 in human midfetal brain, reinforcing that CHD8 targets other regulatory genes *in vivo*. In contrast, genes bound by CHD8 only in hNSCs, and not in human or mouse brain, were enriched for genes containing zinc finger domains or involved in extracellular matrix functions.

### CHD8 depletion causes ASD risk gene dysregulation in hNSCs

**Figure 4:**
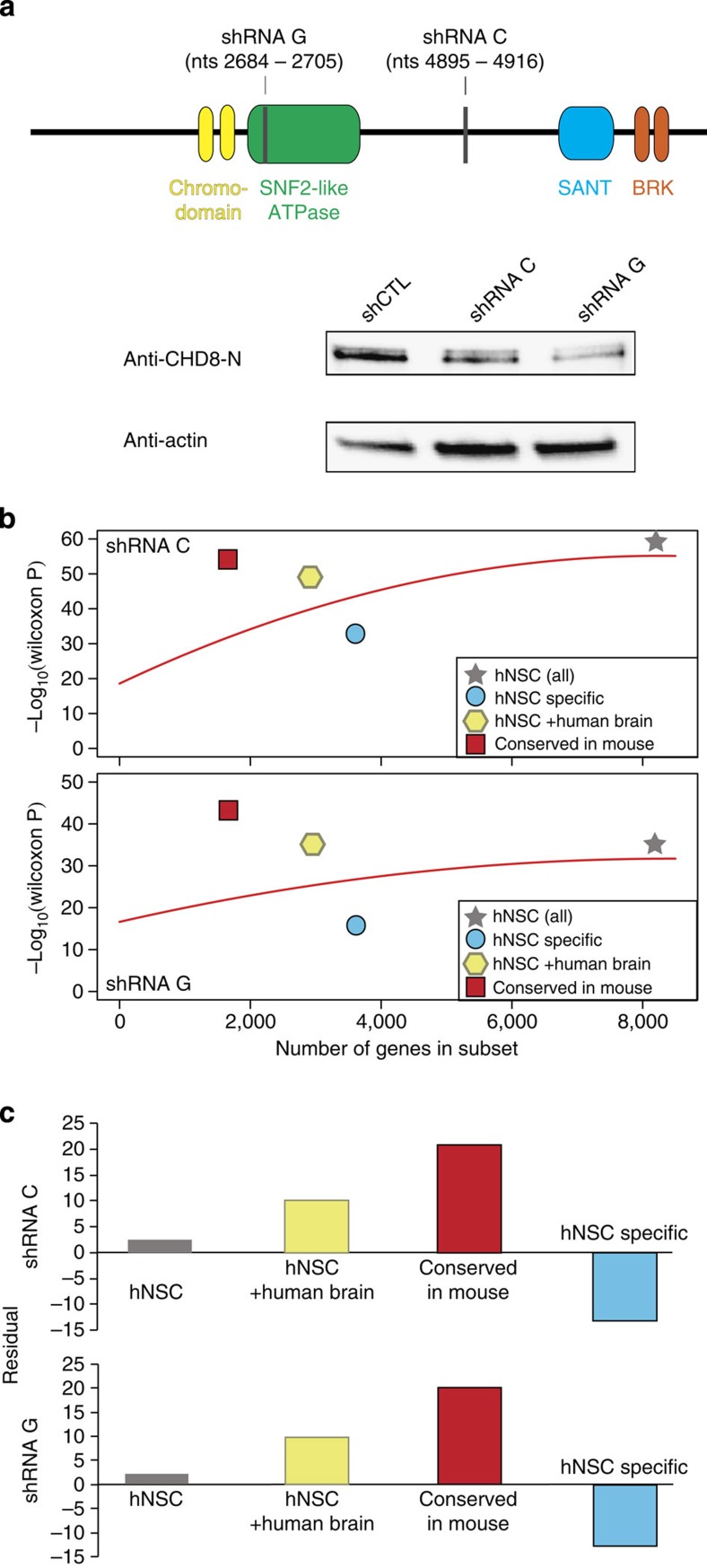
Depletion of CHD8 in hNSCs significantly affects CHD8 target genes. (**a**) *Top*, Schematic depicting functional domains within CHD8. Sites in CHD8 that are targeted by knockdown shRNA constructs C and G are indicated by vertical grey bars. *Bottom*, Representative western blot of hNSC protein lysates demonstrating depletion of CHD8 protein levels due to transfection of each shRNA construct compared with a non-targeting transfection control (shCTL). QPCR and western blots were performed for each knockdown experiment. (**b**) Conserved CHD8 targets are disproportionately affected by CHD8 depletion. For each subset of CHD8 target genes shown, the *P* value from a Wilcoxon rank test comparing the distribution of differential expression *P* values in that subset versus active genes not bound by CHD8 in hNSC is plotted on the *y* axis, and the number of genes in the subset is plotted on the *x* axis (Supplementary Information). The red curve shows the smoothed (quadratic) spline fit to the data. (**c**) Residual values for the indicated subsets of CHD8 targets calculated from the fit lines in **b**. The set of CHD8 targets conserved in mouse holds the greatest fraction of genes showing differential expression by each CHD8 knockdown.

ASD-associated *de novo* truncating mutations in *CHD8* are likely to result in reduced levels of functional CHD8 proteins *in vivo*. To model this putative haploinsufficiency, we carried out knockdowns of *CHD8* transcript levels in hNSCs using two independent short hairpin RNA (shRNA) constructs (Fig. 4a). Both western and quantitative PCR (qPCR) analysis confirmed knockdown of CHD8 transcript from each construct 48 h after transfection (Fig. 4a and Supplementary Fig. 7). Genome-wide analysis indicated these CHD8 shRNAs did not show specificity for any other expressed gene in hNSCs. However, they target different regions of the CHD8 gene and may target distinct CHD8 isoforms (Supplementary Data 5). The shRNAs may thus have different biological effects so we analysed each knockdown independently.

To determine the impact of CHD8 knockdown on gene expression, we performed a series of gene set enrichment analyses^29^. We first compared the distribution of differential expression *P* values from subsets of CHD8 target genes versus active genes not bound by CHD8 in hNSC (Supplementary Information). We plotted the Wilcoxon test *P* value for each subset of CHD8-bound promoters against the number of genes in each set and fitted a smoothed (quadratic) spline to the data (Fig. 4b and Supplementary Data 6 and 7). The residuals from the fitted lines reveal that the set of conserved CHD8 targets holds the greatest fraction of genes showing differential expression by each CHD8 knockdown. In contrast, genes bound by CHD8 specifically in hNSCs, and not human or mouse brain, held a lower fraction of dysregulated genes than expected, as indicated by negative residual values in both knockdowns (Fig. 4c and Supplementary Data 6 and 7). Consistent with these results, conserved CHD8-binding sites exhibit the strongest levels of CHD8 signal in hNSC, suggesting they are robust direct targets of CHD8 regulation (Supplementary Fig. 8). Therefore, depletion of CHD8 in this system results in substantially greater dysregulation of CHD8 targets shared in multiple developmental contexts than of cell-type-specific targets.

To identify biological functions and pathways affected by CHD8 knockdown, we performed gene set enrichment analysis using Kyoto Encyclopedia of Genes and Genomes biological pathways. Pathways showing notable differential expression (Wilcoxon *P*<0.001) in both CHD8 knockdowns included cell cycle, p53 signalling and Hippo signalling (Supplementary Fig. 9). Notably, the cell cycle pathway includes many chromatin interacting proteins, remodellers and modifiers, including the histone acetyltransferases EP300 and CREBBP, the histone deacetylase HDAC1, members of the cohesin complex that regulates chromatin organization (SMC1A, SMC3 and RAD21), as well as the DNA helicase MCM2–7. The p53 and Hippo signalling pathways are known to influence Wnt signalling, which has been previously shown to be targeted by CHD8 (refs 17, 21). Genes that showed the strongest differential expression due to CHD8 knockdown (EdgeR Poisson *P* value<1.68 × 10^−6^ and absolute log_2_ fold change>0.1) were enriched in cell cycle functions, as well as transcriptional regulation, reinforcing the observations obtained from the pathway analysis (Supplementary Data 7).

**Figure 5:**
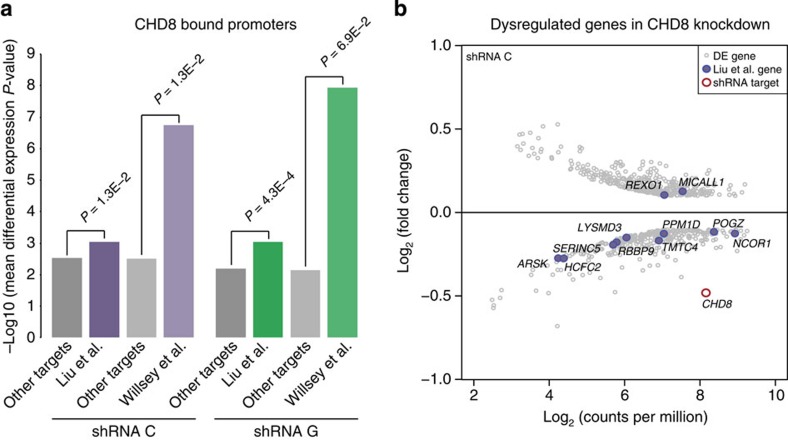
Depletion of CHD8 in hNSCs significantly affects ASD risk genes. (**a**) Mean differential expression *P* values for ASD risk genes from Liu *et al*. or Willsey *et al*. bound by CHD8 versus other genes bound by CHD8 but not in the respective ASD risk gene list. The significance of differences between mean differential expression *P*-values across gene sets was assessed using Wilcoxon rank tests. Note that CHD8 targets in Liu *et al*. are significantly dysregulated compared with other CHD8 targets in both knockdowns, whereas CHD8 targets in Willsey *et al*. are significantly dysregulated compared with other targets only in knockdown C. (**b**) Scatterplot of log_2_ fold change gene expression values and log_2_ read counts per million (CPM) for genes strongly dysregulated in hNSCs transfected with shRNA target C, as compared with scrambled control (EdgeR Poisson *P* value<1.68 × 10^−6^ and absolute log_2_ fold change>0.1, and log_2_(CPM) between 2 and 10). *CHD8* is indicated by a red circle. ASD risk genes from Liu *et al*. are indicated by purple dots.

Finally, we evaluated the effect of CHD8 knockdown on the two sets of ASD risk genes described above. These genes are significantly overrepresented only in CHD8 targets that are shared across multiple neurodevelopmental targets, which is the same CHD8 target set most impacted by CHD8 knockdown and with the greatest CHD8-binding signal. In light of these results, we hypothesized that ASD risk gene expression would be disproportionately affected by CHD8 knockdown compared with other CHD8 gene targets in hNSCs. The overall effect of CHD8 loss on the expression of both sets of genes was generally consistent, in that they were significantly perturbed as a group in at least one knockdown (Supplementary Data 7). Strikingly, we observed that ASD risk genes whose promoters are bound by CHD8 in hNSCs appear to be more significantly dysregulated than other CHD8 targets in these cells. (Fig. 5a). When we considered genes that showed the strongest dysregulation due to CHD8 knockdown, we found that ASD risk genes tended to be downregulated (Fig. 5b). These results, coupled with the co-occurrence of activating chromatin marks at CHD8-bound promoters, suggest CHD8 directly influences the activation of other ASD risk genes in human neurodevelopment.

### CHD8-binding data improves ASD-risk gene detection

**Figure 6:**
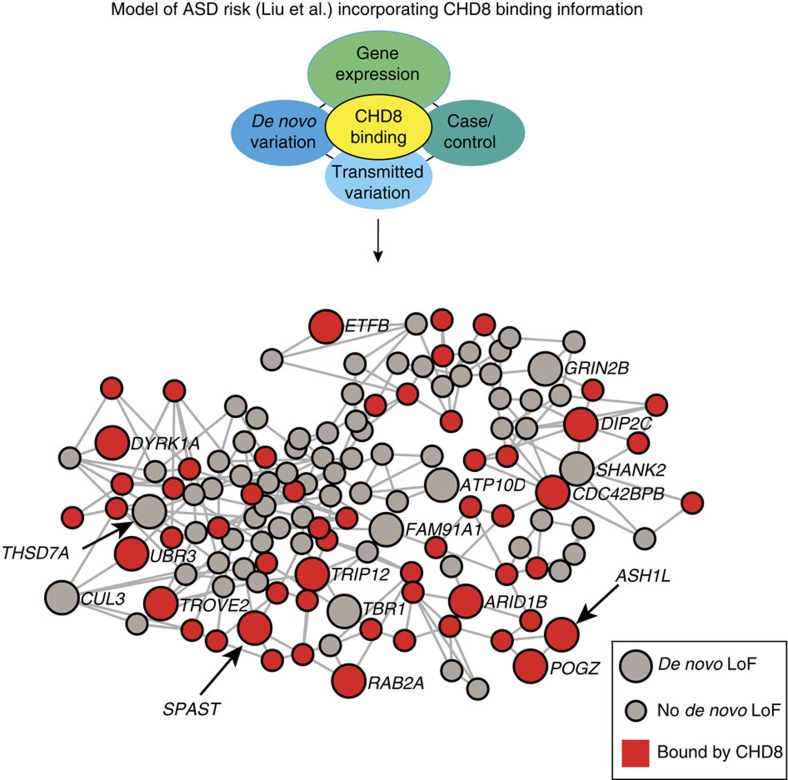
ASD risk network predicted by DAWN model incorporating CHD8 binding. The displayed genes meet the false discovery rate (FDR) threshold of 0.05 based on analysis of the DAWN algorithm^24^ incorporating scoring of *CHD8* binding that is conserved in hNSC and human brain tissues. Red nodes represent genes that are regulated by *CHD8*. Large nodes depict genes that have at least one *de novo* loss of function mutation. *THSD7A, SPAST* and *ASH1L* are new ASD risk genes discovered only after incorporating *CHD8*-binding sites information into the DAWN algorithm. Edges connect genes with absolute partial correlation greater than 0 based on gene expression levels in the midfetal PFC-MSC.

The strong enrichment of ASD risk genes among CHD8 targets indicates that CHD8 binding may provide additional predictive power to identify genes harbouring risk for ASD. To evaluate this, we integrated CHD8 binding at promoters (parameter *d*, see Supplementary Information) into the statistical model initially used to identify the Liu *et al*. ASD risk gene set. We found that CHD8-binding events shared between hNSCs and human midfetal brain significantly increased the discrimination of ASD risk (*d*=1.63, *P*<0.001, Fig. 6 and Supplementary Data 8). Notably, the addition of CHD8-binding information to the model predicted three ASD risk genes that were not detected by the previous implementation of the model (*ASH1L, SPAST* and *THSD7A)*, which incorporated only genetic and gene co-expression data (Fig. 6). CHD8-binding events conserved between human and mouse also provided additional support for ASD risk gene prediction (*d*=1.58, *P*<0.006, Supplementary Data 8). However, CHD8-binding events specific to hNSC did not increase the ability of the model to detect ASD risk genes (*d*=0, *P*=1). Together with our previous results, this reinforces the concept that genes targeted by CHD8 across multiple human neurodevelopmental states, and conserved in mouse, are most likely to include genes conferring risk for ASD.

## Discussion

Our study provides *in vivo*, genome-wide insight into CHD8 binding in human neurodevelopmental tissues, at a developmental stage predicted to be important for ASD aetiology. We provide multiple lines of functional genomics data supporting that CHD8 directly regulates a highly conserved set of targets in human and mouse neurodevelopment. We observed a striking degree of convergence between conserved CHD8 binding and ASD risk, both in the number of known risk genes directly targeted by CHD8 and the disproportionate dysregulation of those genes due to CHD8 knockdown. The substantial ASD risk associated with deleterious *CHD8* mutations may thus reflect a critical role for CHD8 in regulating other potential ASD risk genes in the developing brain. We anticipate that additional ASD risk genes remain to be discovered in the set of CHD8 targets we identified. Supporting this hypothesis, a recent study identified 19 novel high-confidence ASD risk genes exhibiting multiple *de novo* loss of function mutations^30^, 15 of which (79%) are conserved CHD8 targets.

Our results also suggest that loss of CHD8-mediated regulatory control may perturb normal proliferation and differentiation of neuronal progenitors, given the functions of the genes strongly affected by CHD8 knockdown in hNSCs. This may result in altered numbers or relative proportions of neuronal populations derived later in cortical development. Notably, genes directly targeted by CHD8 in multiple tissues and across species showed the greatest risk for ASD. Many of these genes are chromatin modifiers, with known or putative pleiotropic functions. Disruptions in CHD8-mediated regulation due to *CHD8* haploinsufficiency may thus result in phenotypes in addition to ASD, as has been suggested by a recent analysis of over a dozen individuals who carry *de novo* truncating *CHD8* mutations^15^. Identifying the targets of additional chromatin modifiers and transcription factors potentially associated with ASD, and determining how those targets intersect with the CHD8 targets described here, will further reveal the regulatory mechanisms and biological circuitry underlying ASD pathogenesis.

## Methods

### Cell culture

GIBCO Human Neural Stem Cells (H9-Derived) were commercially available from Life Science Technology (N7800100). hNSCs were maintained as recommended per the manufacturer’s protocol. In brief, hNSCs were plated at a seeding density of 5.0 × 10^4^ cells per cm^2^ on a BD Matrigel hESC-qualified Matrix (354277, BD)-coated tissue culture plate, and were cultured in StemPro NSC SFM complete medium that consists of KnockOut D-MEM/F12 medium (12660-012, Life Science Technology), StemPro supplement (A1050801, Life Science), 20 ng ml^−1^ basic FGF recombinant protein (GF003, EMD Millipore Corporation) and 20 ng ml^−1^ EGF recombinant protein (GF144, EMD Millipore Corporation). Cells were incubated at 37 °C, 5% CO_2_ and 90% humidity, and were passaged when they reached 90% confluency (approximately every 3–4 days). hNSCs used in all experiments were passaged five to ten times.

HeLa cells were maintained in DMEM/F12 medium (Life Science Technology) supplemented with 10% fetal bovine serum, 100 U ml^−1^ penicillin and 100 mg ml^−1^ streptomycin, and cells were cultured in a 37 °C incubator with 5% CO_2_.

### Antibody specifications

Antibodies used in westerns and immunoprecipitation (IP)-westerns: anti-CHD8 N-terminal antibody (ab114126, Abcam), anti-CHD8 C-terminal antibody (11891S, Cell Signaling), rabbit purified IgG (3900S, Cell Signaling), anti-actin antibody (ab3280, Abcam), horseradish peroxidase (HRP)-conjugated donkey anti-rabbit secondary antibody (NA934, GE), HRP-conjugated donkey anti-mouse secondary antibody (NA931VS, GE). Antibodies used in ChIP: anti-CHD8 N-terminal antibody (ab114126, Abcam) and anti-H3K27ac (ab4729, Abcam).

### Western blot

Whole-cell extracts from HeLa cells were obtained by lysing the cells in lysis buffer 1 (50 mM Tris, pH 8.0, 140 mM NaCl, 1 mM EDTA, 10% glycerol, 0.5% NP-40, 0.25% Triton X-100, 5 mM dithiothreitol (DTT), 1 mM phenylmethylsulphonyl fluoride (PMSF) and protease inhibitor cocktail (Roche)). For hNSCs, whole-cell extracts were obtained by lysing cells in lysis buffer 2 (20 mM Tris pH 8.0, 150 mM NaCl, 1 mM EDTA, 1% NP-40, 1% sodium deoxycholate, 5 mM DTT, 1 mM PMSF and protease inhibitor cocktail) followed by 2 min sonication. Whole-cell extracts were mixed with Laemmli sample buffer containing 5% β-mercaptoethanol freshly added. Proteins were then separated on a 4–15% SDS-PAGE gel (Bio-Rad). For CHD8 western blots, membranes were incubated overnight at 4 °C with gentle shaking with anti-CHD8 primary antibodies diluted 1,000 times in 5% (w/v) BSA, 1 × Tris Buffered Saline (TBS), 0.1% Tween-20, followed by incubation in HRP-conjugated donkey anti-rabbit secondary antibody diluted 1:10,000 (NA934, GE) for 1 h at room temperature. For actin western blot, anti-actin antibody was diluted 4,000 times in 5% (w/v) non-fat dry milk, 1 × TBS, 0.1% Tween-20, followed by HRP-conjugated donkey anti-mouse secondary antibody (NA931VS, GE). Membranes were visualized using ECL Plus reagents (GE Healthcare). Actin was used as a negative control to measure the decreased expression level of CHD8 in shRNA experiments. Full images of all blots are shown in Supplementary Fig. 10.

### Immunoprecipitation assays

HeLa cells were harvested in cold 1 × PBS and lysed in lysis buffer 1 for 15 min on ice. After centrifugation, the pellet was resuspended in 1 × RIPA buffer (50 mM Tris, pH 8.0, 150 mM NaCl, 1% NP-40, 0.5% sodium deoxycholate, 0.1% SDS, 2 mM EDTA, 10% glycerol, 5 mM DTT, 1 mM PMSF) for 30 min on ice and then sonicated briefly. After centrifugation, the supernatants were kept as nuclear fractions. A measure of 600 μg of the nuclear extracts were incubated overnight at 4 °C with 50 μl of Dynabeads bound to the appropriate antibody (10 μg of CHD8 N-terminal antibody ab114126, 5.6 μg of CHD-8 C-terminal antibody 11891S, 10 or 5.6 μg Rabbit purified IgG). Rabbit purified IgG (3900S, Cell Signaling) was used as a negative control. Fifty micrograms of the nuclear extracts were set aside as input samples. Immunoprecipitates were washed five times with IP washing buffer (1 × PBS, 0.02% Tween-20). Beads were boiled for 5 min at 95 °C in 50 μl of 2 × Laemmli sample buffer containing 10% β-mercaptoethanol to elute proteins. Eluted proteins and input samples were then separated on a 7.5% SDS–PAGE gel (Bio-Rad). Western blots were detected with anti-CHD8 C-terminal primary antibody (11891S, Cell Signaling) and HRP-conjugated donkey anti-rabbit secondary antibody (NA934, GE) as indicated above.

### Chromatin immunoprecipitation

C57B6/J mice were housed and killed as per Yale IACUC protocols. Mouse embryonic cortical tissue was dissected at E17.5 and briefly homogenized in cold PBS. Tissue was crosslinked with 1% formaldehyde at room temperature for 15 min with rotation, followed by quenching with 150 mM glycine. Use of human fetal tissue was reviewed and approved by the HIC committee of the Human Research Protection Program at the Yale University. Human tissue was collected after appropriate informed consent by the Department of Neurobiology at the Yale School of Medicine in accordance with ethical guidelines and regulations for the research use of human brain tissue set forth by the NIH (http://bioethics.od.nih.gov/humantissue.html) and the WMA Declaration of Helsinki (http://www.wma.net/en/30publications/10policies/b3/index.html). Human Period 5 (16–19 PCWs) fetal brain tissue was dissected from the striatum, cerebellum (CBC), primary visual cortex (V1C) and dorsal frontal cortex (DFC) of two different specimens as described for the Brainspan Brain transcriptome^31^ (brainspan.org). Samples were thawed in PBS and homogenized, crosslinked with 1% formaldehyde, then quenched with 150 mM glycine. hNSCs were washed twice with PBS while still adherent. PBS was removed and 10 ml of PBS containing 1% formaldehyde was added directly to the plate. Crosslinking occurred for 15 min at room temperature with rocking every 3 min, then was quenched with addition of glycine (150 mM final). hNSCs were scraped from the plate, collected in 15 ml conical tubes and harvested by centrifugation. All tissue and cell pellets were washed with PBS and flash-frozen for subsequent nuclear extraction and lysis. Isolation of nuclei, extraction of chromatin and shearing with sonication were carried out as previously described^32^. Chromatin was immunoprecipitated by incubating 15–100 μg of soluble chromatin with 10 μg of CHD8 antibody (Abcam, ab114126) or 20 μg of chromatin with 2 μg of H3K27ac antibody (Abcam, ab4729) prebound to Protein G Dynabeads (Invitrogen) overnight at 4 °C. CHD8-bound beads were washed five times with 500 mM NaCl IP wash buffer and once with Tris EDTA, whereas H3K27ac-bound beads were washed eight times with 500 mM LiCl IP wash buffer and once with TE. Harvested chromatin was then eluted from the beads, crosslinks were reversed and DNA was purified as previously described^32^. All samples were prepared for sequencing by the Yale Center for Genome Analysis. An input library was prepared in parallel using chromatin that was sonicated but not immunoprecipitated. The libraries were sequenced on Illumina HiSeq 2500 (75 bp SE reads).

ChIP-seq reads were aligned to the mm9 and hg19 reference genomes using Bowtie (v0.12.9)^33^, and uniquely aligned reads were kept for further analysis (−m 1 option). A sliding window approach was used to call peaks of enrichment with a *P* value cutoff ≤10^−5^ as previously described^32^. All mouse peaks were converted to hg19 coordinates using liftOver and chain files from the UCSC source tools^34^. Promoter (within 1-kb upstream of the TSS), exon and enhancer peaks were identified based on the ENSEMBL v72 gene annotation using BedTools^35^. One-to-one orthologous genes between human and mouse were retrieved using Ensembl BioMart. ChIP-seq fragment densities were generated by extending each aligned read to 300 bp based on sonication size then counting the number of extended fragments that overlap each nucleotide. Read counts were then normalized to fragments per million aligned reads. Reproducible enriched regions in hNSCs or mouse E17.5 cortex were defined as those that had 1 bp minimum overlap between two biological ChIP-seq replicates. For CHD8-bound regions in human brain, reproducibility was defined as overlapping peaks from any two replicates of cortical regions (V1C and DFC) or non-cortical regions (CBC and Striatum). Merged coordinates from both replicates were then used to define a reproducible region. Gene ontology analysis of CHD8-bound promoters was performed using DAVID^36^. Additional hNSC ChIP-Seq data sets were retrieved from the RoadMap Epigenome Project (http:// commonfund.nih.gov/epigenomics/; ftp://ftp.ncbi.nlm.nih.gov/pub/geo/DATA/roadmapepigenomics/by_sample/H1_derived_neuronal_progenitor_cultured_cells/).

### Motif enrichment

CHD8-binding sites identified as conserved between human tissues and mouse and overlapping gene promoters were selected for analysis. The centre of each peak was identified and a 200-bp window of DNA sequence was extracted using BedTools. Resulting DNA sequences were processed for motif enrichment and matching to known transcription factor-binding sites using Homer (v4.6)^37^ and DREME/TOMTOM (v4.9.0)^38^.

### shRNA knockdown

The following GIPZ lentiviral shRNA constructs were used in the current study: a scrambled control shRNA construct (RHS4346, GE), CHD8 shRNA C (CloneID: V2LHS_201084, GE), mature antisense sequence 5′-TAAAGACTCCAATGAGCAG-3′); CHD8 shRNA G (Clone ID: V3LHS_311510, GE), mature antisense sequence 5′-ACTGTTGAATCATCTGCCT-3′). Each shRNA construct includes the TurboGFP reporter gene driven by a human CMV promoter for convenient tracking of expression and selection by flow cytometry. 1.0 × 10^6^ hNSCs were transfected with 1 μg of constructs encoding the scrambled control shRNA, CHD8 shRNA C or CHD8 shRNA G, respectively, using Amaxa Mouse NSC Nucleofector Kit (VPG-1004, Lonza), programme A-033.. Cells were grown for 48 h in KnockOut DMEM/F-12 medium before sorting. The scrambled control shRNA was used as a baseline to measure the specific knockdown effects for any transfection experiment performed using CHD8 shRNA constructs. Each shRNA knockdown experiment included the scrambled control shRNA, CHD8 shRNA C and CHD8 shRNA G and were performed in quadruplicate. Two replicates were transfected and sorted on the same day, the other two replicates were done on two different days. Both western blot and reverse transcription (RT)-qPCR were used to determine the knockdown efficiency of CHD8 before performing RNA-seq.

### Fluorescence-activated cell sorting (FACS)

Forty-eight hours after transfection, hNSCs expressing each of the above shRNA constructs were dissociated by Accutase (Life Science Technology), gently resuspended in KnockOut DMEM/F-12 medium, and filtered through a 35-μm nylon mesh (BD Biosciences). All samples were kept on ice before sorting. Cells were sorted at a rate of ∼3,000 events per second on a fluorescence-activated cell sorter FACSAria II (BD Biosciences). Digital data were collected using FACS Diva software (BD Biosciences). Before sorting, the nozzle, sheath and sample lines were sterilized with 70% ethanol and Diethylpyrocarbonate (DEPC)-treated water. Between running two samples, the system was cleaned with DEPC-treated water. Both 80 μm and 100 μm nozzles (BD Biosciences) were used for hNSC cell sorting. Forward-angle and side-angle light scatter were used to set the gate for live cells. Green fluorescent protein (GFP) fluorescence intensity was detected using a blue laser operating at 488 nm and a 530/30 nm band-pass filter for fluorescein isothiocyanate/GFP. hNSCs not expressing GFP were used to determine the threshold parameters for selecting cell populations with GFP signals. Sorted cells were collected in KnockOut DMEM/F-12 medium, spun down, resuspended in 700 μl QIAzol (Qiagen) and stored at −80 °C for downstream analysis.

### RNA isolation and RT-qPCR

Total RNA was extracted using the miRNeasy Micro Kit with on-column DNase digestion (217084, Qiagen), as described in the manufacturer’s instructions. cDNA was generated from 10 to 25 ng of total RNA using Superscript III First Strand Synthesis Supermix (18080-400, Invitrogen); random hexamer primers were used for cDNA synthesis. A measure of 250 pg of cDNA were used as template for RT-qPCR in a 20-μl reaction containing 1 × PowerSybr Master Mix (ABI) and 1.25 μM Primers. Ct values were determined in triplicate on an ABI StepOnePlus instrument. Ct values were normalized to the expression of the housekeeping gene *ACTB*, and ΔΔCt values were utilized in detecting CHD8 expression differences. Primers were designed using Primer3 plus. The primer sequences are:

*CHD8* (exon 4–5) forward primer (5′- CTGCACAGTCACCTCGAGAA -3′)

*CHD8* (exon 4–5) reverse primer (5′- TGGTTCTTGCACTGGTTCAG -3′)

*CHD8* (exon 36–37) forward primer (5′- TGAACTGTTTGGGAATGGAA -3′)

*CHD8* (exon 36–37) reverse primer (5′- TGCTGCTCTCTGGTGCAATA -3′)

*ACTB* forward primer (5′- GGCATCCTCACCCTGAAGTA -3′)

*ACTB* reverse primer (5′- AGCACTGTGTTGGCGTACAG -3′).

### Enrichment of ASD risk genes within CHD8 target genes

To determine whether CHD8 promoter targets were enriched with ASD risk genes, we considered the overlap between the 127 ASD genes discovered by *de novo* mutations from exome sequencing from Liu *et al*. and the CHD8 active promoter targets. Four lists of genes were compared using Ensembl 75 gene definitions: (i) 11,267 active promoters, defined as having at least one active chromatin mark (H3K27ac or H3K4me3) in hNSCs and as being on the list of 20,759 genes targeted by exome capture; (ii) 127 ASD genes and (iii) CHD8-targeted promoters (the number of these varied according the tissue used for the ChIP-Seq). Of the 127 ASD genes, 117 were in active promoters and these were used to assess the degree of CHD8 target enrichment.

### Gene permutation for ASD genes

To assess the enrichment of CHD8 active promoter targets in the 117 ASD genes in active promoters, we performed a permutation test by permuting the identity of the 117 ASD genes and assessing the fraction of these genes that were also CHD8-active promoter targets.

The total size of the coding exons in each gene, the GC content and the exome coverage are all known confounders of *de novo* mutation rate, therefore the permutation test was designed to account for these confounders. Size was determined by the total number of coding nucleotides covered by at least one gene isoform in Ensembl 75. The percentage of GC content was estimated from the hg19 reference for each gene. Finally, the percentage of nucleotides with at least 20 unique reads in the exome data was estimated for each locus identified in the assessment of gene size, compared with ten representative BAM files (randomly chosen from a list of BAM files used to identify the *de novo* mutations used as the input in Liu *et al*. and excluding files that differed in size (bytes) from the mean by more than one standard deviation); the median percentage of nucleotides at each locus from the ten BAM files was used.

The mutability of each gene was estimated by:




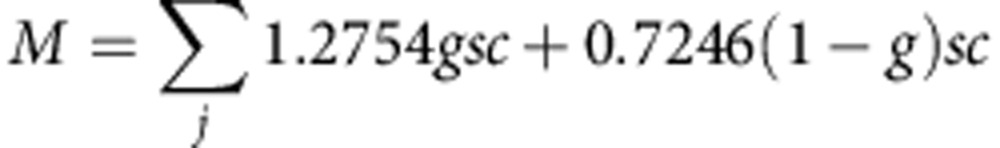




Where *M* is the mutability of the gene; *j* is the number of discrete loci (exons) in the gene; *g* is the percentage of GC content in each discrete locus; *s* is the number of nucleotides in the discrete locus and *c* is the fraction of the discrete locus with at least 20 unique reads (estimated as the median of ten representative samples). The constants of 1.2754 and 0.7246 represent the change in the expected rate of mutability for GC nucleotides and AT nucleotides, respectively^39^.

To simulate an ASD gene, the cumulative mutability was calculated from all genes under consideration and a random number was generated between zero and the total sum of mutability; the gene with a cumulative mutability corresponding to this random number was selected. For each iteration this gene selection process was repeated until 117 genes had been selected. A gene could only be selected once in each iteration. The number of these 117 genes that were also CHD8-active promoter targets was recorded and the *P* value estimated as the number of iterations with the greater than or equal to the observed number of ASD genes that were CHD8-active promoter targets over the total number of iterations.

### Promoter permutation for CHD8 promoters

As an alternative strategy to estimating the significance of enrichment for CHD8 targets in ASD genes, we permuted the CHD8 target promoters rather than the ASD genes. Promoters were defined as the 1,000 nucleotides immediately upstream of the transcription start site. The promoter region of a gene can differ between isoforms, therefore a gene with multiple isoforms potentially has a larger total promoter size than a gene with a single isoform. Since many brain-expressed genes have multiple isoforms, this might bias our assessment of the significance of CHD8 target enrichment in ASD genes. To control for this potential bias, we calculated the total number of nucleotides that were identified as promoters by at least one isoform of each gene. This promoter size was estimated for every gene based on Ensembl 75.

Similar to the mutability measure used for ASD mutations, the cumulative promoter size was calculated from all 11,267 active promoters under consideration and a random number was generated between zero and the total sum of promoter sizes; the gene with a cumulative promoter size corresponding to this random number was selected. For each iteration of the permutation test, this process was repeated until the desired number of promoters was achieved (based on the number of CHD8-active promoters targets). A gene could only be selected once in each iteration.

The number of these permuted CHD8 promoters that were also ASD genes was recorded and the *P* value estimated as the number of iterations with the greater than or equal to the observed number of ASD genes that were CHD8-active promoter targets over the total number of iterations.

### Enrichment analyses using *de novo* ASD risk genes

The methods described above were repeated using the 116 pASD risk genes from Willsey *et al*. instead of the 127 ASD genes from Liu *et al*. As before, the genes were compared using Ensembl 75 gene definitions. The pASD genes were permuted using the same model of identifying random genes based on gene size, GC content and exome coverage. The promoters were permuted as described above.

### Construction of spatiotemporal co-expression networks

Gene co-expression networks were constructed as previously described^13^. However, to ensure that any observed enrichment of CHD8 promoter targets was not driven by overrepresentation of active genes within the co-expression networks (CHD8 peaks are strongly associated with active genes—see Supplementary Fig. 2), the background set of 16,947 genes was further trimmed to those with Ensembl 72 gene definitions and promoters with active histone marks in hNSCs (11,267 genes). hcASD and ASD risk gene lists were also trimmed according to the same criteria.

This analysis focused on two spatiotemporal networks previously associated with ASD in Willsey *et al*.^13^: the period 3–5 and period 4–6 PFC-MSC networks. After construction, each of the co-expression networks were assessed by permutation test (10,000 iterations) for enrichment of both ASD risk genes^13^ and CHD8 promoter targets.

### Permutation tests of gene and promoter target enrichment

Permutation tests were also conducted as in Willsey *et al*.^13^ Specifically, for the previous analysis ‘Enrichment of ASD risk genes within CHD8 target genes,’ 10,000 sets of hcASD genes were permuted. These genes were utilized as seeds for construction of 10,000 null co-expression networks. The significance of the observed gene enrichments was then determined by comparison to the distribution of enrichment among the permuted co-expression networks. hcASD genes (observed or permuted) that were also CHD8 promoter targets were not counted as hits within the observed or permuted co-expression networks.

### RNA-Seq and CHD8 knockdown expression analysis

mRNA purification and preparation of strand-specific sequencing libraries were performed by the Yale Center for Genome Analysis. Samples were sequenced on Illumina HiSeq 2500 instruments (75 bp paired end reads). Reads were mapped to UCSC knownGene (retrieved 05/13/2013) or ENSEMBL v72 using TopHat (v2.0.9)^40^. Read counts per gene were extracted using HTSeq (v0.5.4)^41^ and filtered based on several quality metrics. The initial quality control (QC) step looked at a multidimensional scaling (MDS) plot of the raw reads. Separation of batches occurs by MDS dimension 1, whereas the separation of the treatment does not clearly show until MDS dimension 4. The next QC step was to remove 7,245 genes from the analysis with a total read count across the treatments and batches of ≤20. Many of these genes have zero read counts in most units. An additional four genes were removed from the analysis whose overall read count was all based on one of the experimental units. After this edit, there were 16,461 genes remaining for analysis. The reads were subsequently normalized using the following procedure:

- For each experimental unit *i* determine the size of the library 
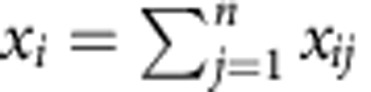
, where *n* is the number of genes and *x*_*ij*_ is the read count for experiment *i* and gene *j*.
- Determine the average of the *i* libraries 
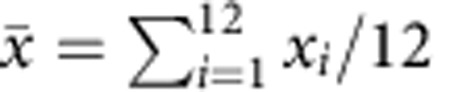
 .
- Calculate the normalized read counts 
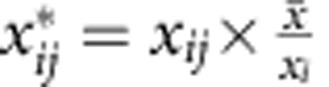
 .

Based on the normalized read counts the Variance to Mean Ratio (VMR) was calculated for each of the genes within each of the treatments. For Poisson distributed count data, VMR is expected to be near 1 and values of >10 rarely occur when drawing 4 random values from a Poisson distribution. Similarly, the VMR for four random samples of a negative binomial with a success rate of 0.05 is generally <100. As a result, we removed 1,620 genes from the analysis for which the maximum VMR value calculated within treatment group exceeded 100 resulting in 14,841 genes remaining for downstream analyses. Data were analysed contrasting knockdown C versus controls and knockdown G versus controls. A Poisson model with batch and knockdown effects was used. Data were normalized using the trimmed mean procedure in edgeR^42^. In addition, the offset was set to the log(x_ij_) using the standard feature in edgeR. Analysing the data in edgeR with a Poisson model was accomplished by setting the dispersion variable in the glmFit function to 0. We also used the edgeR for the analysis of the data using a negative binomial model. This implementation uses a variance shrinkage approach to analysing the data. Again knockdown and batch effects were included in the model and knockdown C and knockdown G were analysed separately, each time contrasting them to the control.

### Analysis of gene sets

Results from the Poisson and negative binomial models were used to check for enrichment of small *P*-value genes in subsets of genes based on functional criteria: all genes with active promoters from H3K27ac data, all CHD8-bound promoters in hNSCs, CHD8-bound promoters shared between hNSC and human brain, CHD8-bound promoters conserved between human and mouse, and CHD8-bound promoters specific to hNSC. Gene names in these lists were matched to *P*-values from the edgeR analysis. We then tested whether the ranks of genes, based on *P*-values, were distributed differently if the genes were included versus excluded for a set of CHD8-bound genes. Significance was determined using a Wilcoxon rank test. To lend further insight into these results, we next plotted the *P*-value for each subset of CHD8-bound promoters against the number of genes in the gene set N and fitted a smoothed (quadratic) spline to the data. The residuals from the fitted lines reveal that the set of conserved CHD8 targets holds the greatest fraction of genes showing differential expression by each CHD8 knockdown.

We next set out to determine whether there is enrichment for sets of genes associated with risk for ASD. We used two sets of genes taken from recent literature^13^,^24^ and filtered them based on presence in above quality-controlled gene expression lists. We tested for distributions of *P*-values in these lists versus background genes using Wilcoxon rank test. Note that we are performing two tests per list to calculate *P* values in Supplementary Data 7 and Fig. 5a, so the significance level is 0.05/2=0.025.

We next turned to analysis of pathways, as defined by KEGG, to determine whether certain pathways of genes show notable differential expression as a result of CHD8 knockdown. Power in this setting is a function of number of genes comprising the pathway. Therefore, we limited the analysis to KEGG pathways that have 20 or more genes characterizing them. This criterion results in 218 pathways tested for impact of the two knockdown constructs. We take the Bonferroni threshold of.05/436=0.00011 as significantly enriched and 0.001 as notable. It is worth noting that the lists are relatively small and therefore the test will be poorly powered unless there is a very large effect. Significance of enrichment in each pathway was determined again by Wilcoxon Rank Test. By this criteria and knockdown C, the cell cycle pathway emerges as significant (*P*=0.000015) and the P53 signalling pathway is notable (*P*=0.00042). For knockdown G, four pathways are significant: cell cycle (*P*=1.21E-05), RNA transport (*P*=2.04E-05), spliceosome (*P*=8.68E-05) and p53_signaling_pathway (*P*=9.38E-05); and four are notable: ribosome (*P*=0.00025), Hippo signalling (*P*=0.00028), DNA replication (*P*=0.00043) and adherens junction (*P*=0.00092).

### Identifying strongly dysregulated genes in CHD8 knockdowns

To judge differential expression, we target two features: significance as measured by the Poisson model and magnitude of differential expression. We take the threshold for differential expression of *P*<1.68 × 10^−6^ as a first cut of the genes. This is the Bonferroni correction for 14,841 tested genes by two knockdown constructs, that is, 0.05/[14,841*2]. Note that genes showing relatively small differential expression can still exceed this significance threshold if their read counts are large. We then require the logFC >0.1, so that the fold change is meaningful. Finally, to exclude genes that do not meet the Poisson assumption or have very high read counts, we restrict the analysis to genes with log counts per million between 2 and 10.

### Prediction of further ASD risk genes using CHD8-binding data

Using a new approach called DAWN, Liu *et al*.^24^ model two kinds of data: rare variations from exome sequencing^43^ and gene co-expression in the mid-fetal prefrontal and motor-somatosensory neocortex, a critical nexus for risk^13^. Using these data, DAWN identified 127 genes that plausibly affect risk.

The DAWN algorithm casts the ensemble data as a hidden Markov random field in which the graph structure is determined by gene co-expression. It combines these interrelationships with node-specific observations, namely gene identity, expression, genetic data and the estimated effect on risk. Here we extend the DAWN approach by incorporating information about binding site targets for CHD8. If the term in the DAWN model that incorporates binding site status into the model is significantly greater than 0, it supports the theory that CHD8-binding sites predict ASD risk status.

The first step of DAWN requires an estimate of the gene network, that is, the adjacency matrix. In Liu *et al*.^24^, the network is estimated using a thresholded version of the correlation matrix. Because the resulting network is quite dense, clusters of highly correlated genes are combined to create multigene nodes. When incorporating information about CHD8-binding sites into the model, however, it is better if each node represents a single gene. For this reason, we modified the original DAWN algorithm to produce a sparse network with single-gene nodes.

We estimate the network using a sparse regression technique to select the non-zero partial correlations. Following Meinshausen and Buhlmann^44^, we apply the lasso to each neighbourhood regression and then construct the adjacency matrix by aggregating the non-zero partial correlation obtained for each regression. Some adjustments were made to this approach to focus on key nodes in the network based on genetic information and pairwise correlations.

To determine the right choice for the smoothing parameter, we rely on the fact that many biological networks follow a power law^45^.

### The DAWN algorithm

Let ***I***=(*I*_1_,…, *I*_*n*_) be a binary vector indicating which genes are associated with ASD. This is the ‘hidden state’. The original DAWN model, *M*_0_, assumes that the distribution of ***I*** follows an Ising model with density




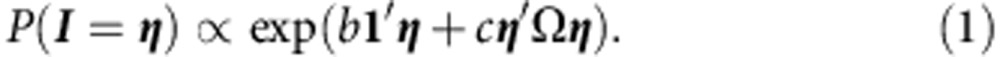




To incorporate the CHD80binding site information, we propose the generalized Ising model, *M*_1_, that incorporates the directed network indicating, which genes are regulated by CHD8. The density function of the generalized Ising model is as follows:




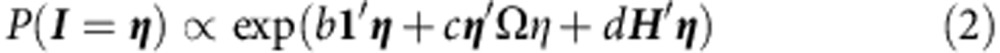




where ***H***=(*h*_1_,…, *h*_*n*_) is the indicator of CHD8-binding sites, and *d*>0 reflects the enhanced probability of risk for genes regulated by the chromatin modifier.

The corresponding *P* values derived from TADA are converted to *Z*-scores (***Z***) to obtain a measure of the evidence of disease association for each gene. It follows immediately that each of the Z-scores under the null hypothesis *I*=0 has a standard normal distribution. We assume that under the alternative *I*=1 the *Z*-scores approximately follow a shifted normal distribution. To fit *M*_0_, we apply the iterative algorithm described in Liu *et al*.^24^ to estimate the parameters of the model. Minor adjustments of the DAWN algorithm permit the estimation of the additional parameter *d* in *M*_1_.

### Testing the CHD8-binding site effect

If *d*>0 this indicates that the CHD8-binding site covariate is a predictor of risk for ASD. To test whether or not 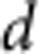 is significantly larger than zero, we compare the observed statistic 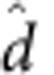 with *d* obtained under the null hypothesis of no association. We do so using a smoothed bootstrap simulation that involves simulating data with the same clustering of genetic signals, but without an association with the CHD8-binding sites.

To simulate ***Z*** from ***M***_0_, we first simulate the hidden states ***I*** from the distribution (1). Initial values of ***I*** are given to each node in the simulated graph, with a proportion of *r* being 0.5. Then, we apply a Metropolis–Hasting algorithm to update ***I*** until convergence:

1. Apply the algorithm to model ***M***_0_ to obtain estimates of the model parameters.
2. Using the estimated null model, simulate ***Î*** by the Metropolis–Hastings algorithm, then simulate 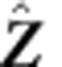.
3. Using model ***M***_1_, estimate the parameters for the simulated data.
4. Iteratively conduct step (2–3) *N* times, then compute the empirical *P* value for *d* by comparing the realized and simulated values.

## Additional information

**How to cite this article:** Cotney, J. *et al*. The autism-associated chromatin modifier CHD8 regulates other autism risk genes during human neurodevelopment. *Nat. Commun.* 6:6404 doi: 10.1038/ncomms7404 (2015).

## Supplementary Material

Supplementary Figures and Supplementary References Supplementary Figures 1-10 and Supplementary References

Supplementary Data 1 Reproducible CHD8 peaks identified in each experimental context and potential target genes. Each sheet contains the coordinates of the CHD8 peak in bed format along with the coordinates of the promoter or regulatory domain for each target gene.

Supplementary Data 2 Homer and Dreme/TomTom detected motif enrichment within 200 bp of CHD8 promoter binding sites shared between hNSC and human brain.

Supplementary Data 3 Permutation results for detecting enrichment of ASD risk genes within CHD8 bound promoters for each subset indicated in the text. For each indicated gene the mRNA size, GC content, coverage, mutability, promoter or enhancer activity (H3K27ac), binary expression level, ASD risk membership, and CHD8 binding status are listed.

Supplementary Data 4 DAVID Gene Ontology analysis of promoters bound by CHD8 in human tissues and mouse cortex.

Supplementary Data 5 Blastn analysis of shRNA constructs C and G to determine specificity for CHD8 transcripts.

Supplementary Data 6 Wilcoxon P-values for subsets of CHD8 bound promoters and residual values from fitting of smoothed (quadratic) spline as shown in Fig 4B.

Supplementary Data 7 Gene expression analysis in CHD8 knockdown experiments. See readme in first sheet for complete details.

Supplementary Data 8 Impact of CHD8 binding on prediction of additional ASD risk genes. For each list of CHD8 peaks, the number of genes assigned, the d value from the modified DAWN algorithm, and permuation P-value are indicated.
