# Supplementary material for: The autism-associated chromatin modifier CHD8 regulates other autism risk genes during human neurodevelopment
Source: Nat Commun. 2015 Mar 10;6:6404. doi: 10.1038/ncomms7404 (PMC4355952; doi:10.1038/ncomms7404)
Supplement: Supplementary Figures and Supplementary References — Supplementary Figures 1-10 and Supplementary References [file ncomms7404-s1.pdf]

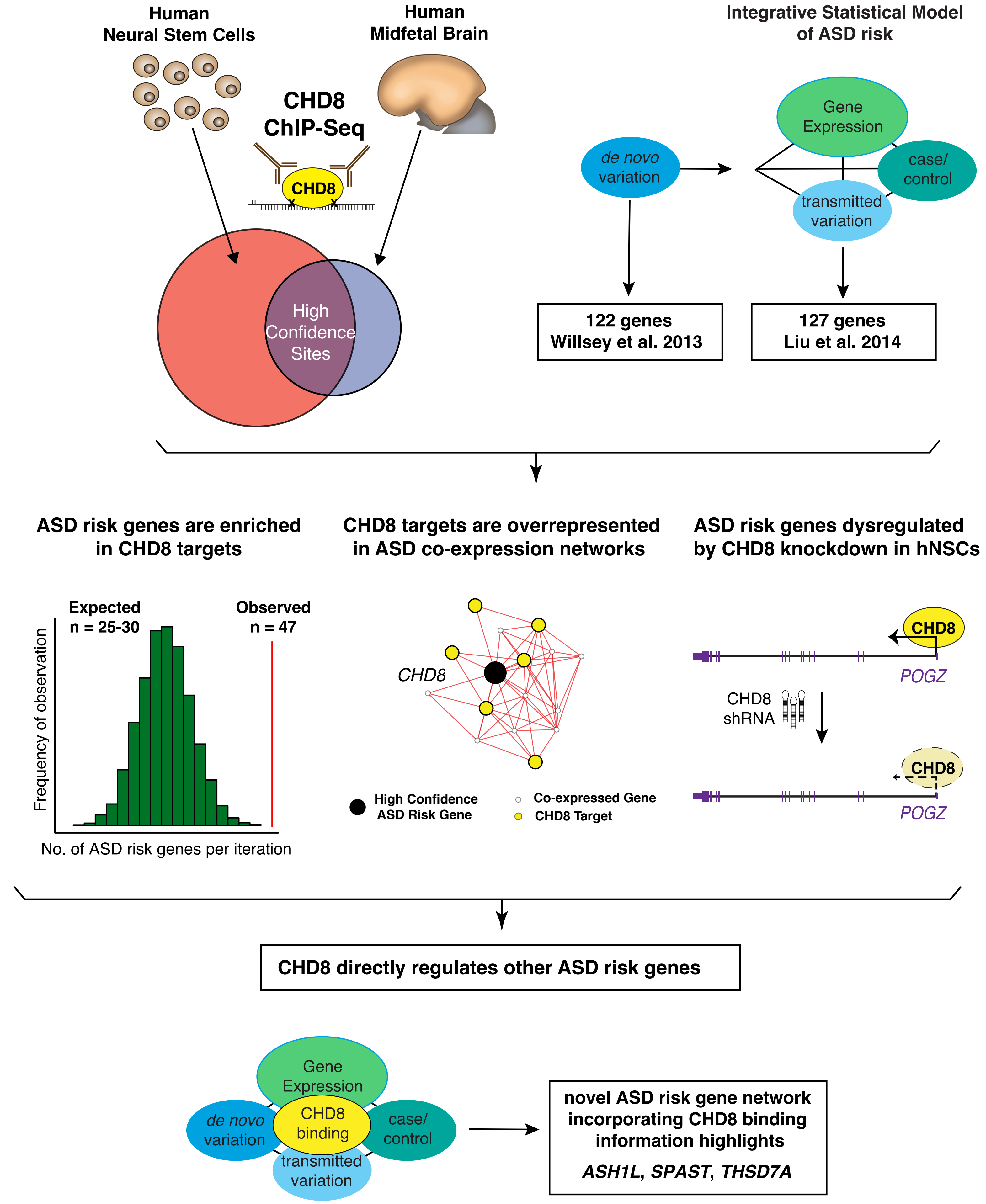

Supplementary Fig. 1. Experimental schema and analyses performed in this study.

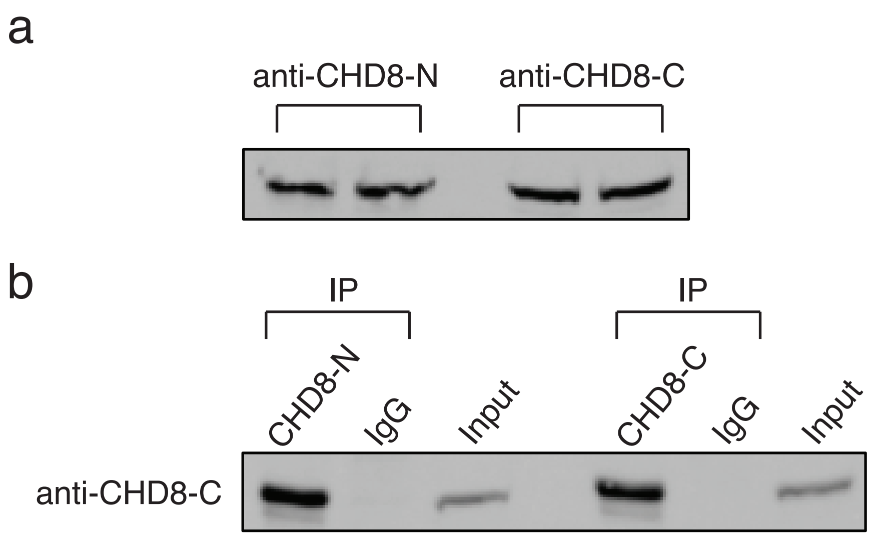

Supplementary Fig. 2. CHD8 antibody validation. a) Representative western blot detecting CHD8 protein isolated from HeLa whole cell extracts. Two independent antibodies targeting either the amino-terminal (CHD8-N, Abcam ab114126) or carboxy-terminal (CHD8-C, Cell Signaling 11891S) portions of CHD8 detect a 290 kDa protein. Duplicate lanes were run on the same protein gel, transferred and incubated separately with primary antibodies as indicated. Secondary antibody binding and chemi-illuminescent detection were performed as indicated in Methods. b) Representative immunoprecipitation of CHD8 from HeLa nuclear extracts with either CHD8-N or CHD8-C antibody and followed by western blotting utilizing the CHD8-C antibody. Immunoprecipitation was performed in parallel with rabbit IgG as a control. Input is 10% of lysate used in immunoprecipitation. Each set of experiments was performed twice.

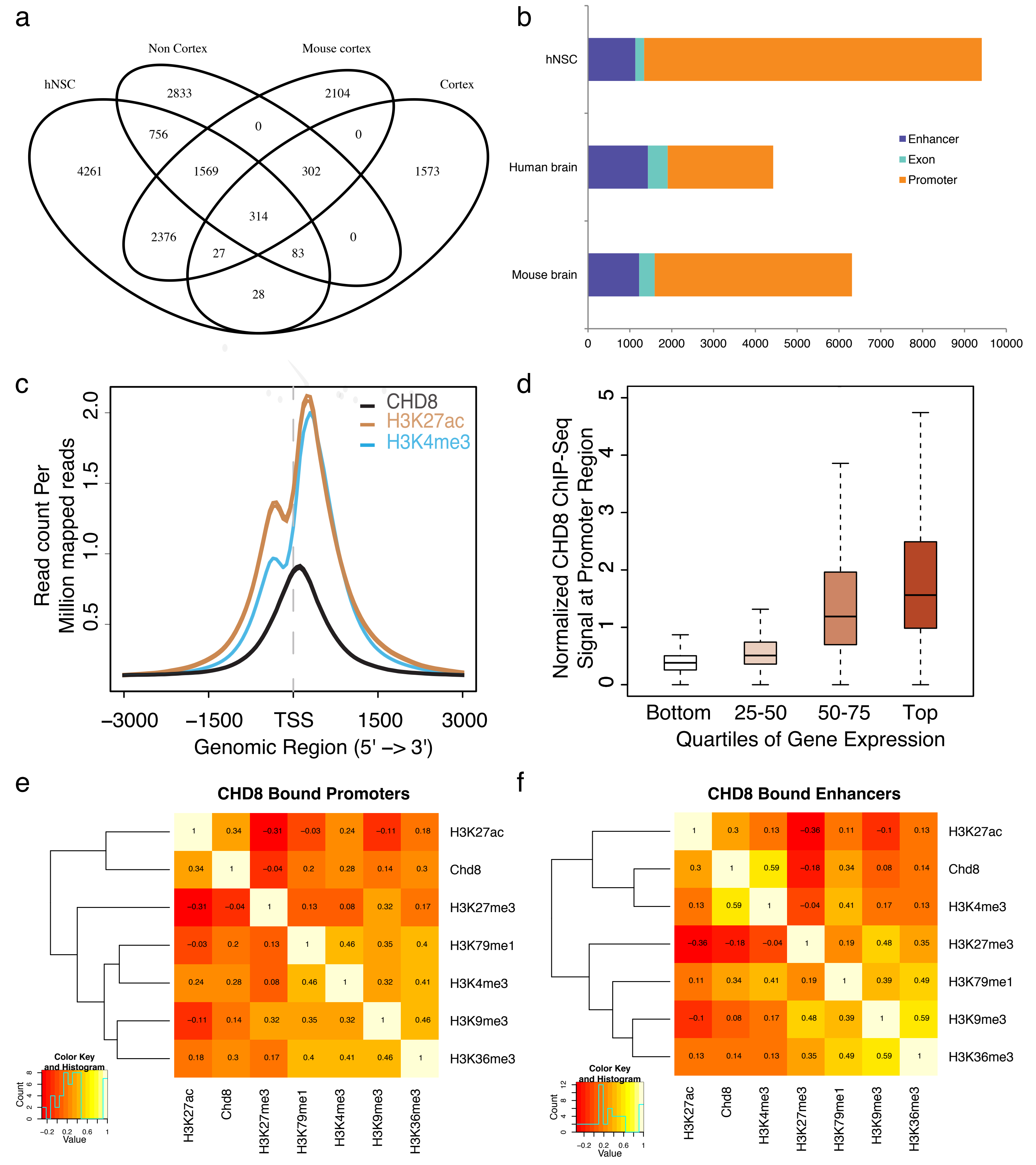

Supplementary Fig. 3. CHD8 binding distribution and co-occurrence with marks. a) Total number of reproducible CHD8 peaks and overlap between datasets for hNSC, cortical and non-cortical regions of human midfetal brain, and mouse E17.5 cortex. b) Numbers of genomic features bound by CHD8 in hNSC, human brain, and mouse E17.5 cortex. c) Aggregate signal of CHD8, H3K4me3, and H3K27ac ChIP-seq signals from hNSCs relative to gene transcription start sites. CHD8 accumulates just downstream of the transcription start site (TSS) and coincides with active chromatin marks. d) Normalized CHD8 ChIP-seq signal surrounding the TSS of genes expressed at increasing levels (in quartiles) in hNSCs. e) Heatmap showing spearman correlation values between CHD8 ChIP-seq signals and indicated chromatin ChIP-seq signals from hNSCs at CHD8 bound promoters. f) Heatmap showing spearman correlation values between CHD8 ChIP-seq signals and indicated chromatin ChIP-seq signals from hNSCs at CHD8 bound putative enhancers.

a

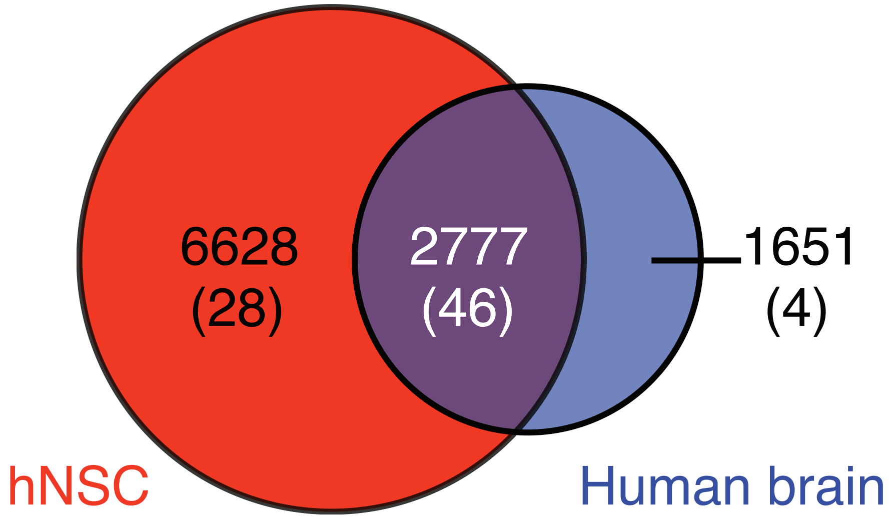

b

ADNP	*FARP1*	*RNF38*
APH1A	*FOXP1*	*SETBP1*
ARID1B	*MED13L*	*SETD2*
ASH1L	*MLL5*	*SMARCC2*
ATP1B1	*MPHOSPH8*	*SPAST*
BRWD1	*MYH10*	*SUV420H1*
CBX4	*NCKAP1*	*TRIP12*
CDC42BPB	*NFKBIL1*	*TROVE2*
CNOT3	*NUAK1*	*UBN2*
CTTNBP2	*PHF2*	*UBR3*
DDX3X	*PHF7*	*USP15*
DIP2A	*PLXNB1*	*VCP*
DIP2C	*PPM1D*	*WDFY3*
DST	*PPP1R15B*	*ZMYM2*
EFCAB5	*RAB2A*	*ZMYND11*
ETFB		

Supplementary Fig. 4. ASD risk genes bound by CHD8 in human neurodevelopment. a) Reproducible CHD8 binding sites identified in two biological replicates of hNSC and midfetal human brain. The number of reproducible sites in each tissue and the subset identified in both tissues are indicated in each section of the Venn diagram. The number of ASD risk genes from Willsey et al. bound by CHD8 in each subset is noted in parentheses. b) List of ASD risk genes identified by Willsey et al. with shared CHD8 binding between hNSCs and midfetal human brain (n=46).

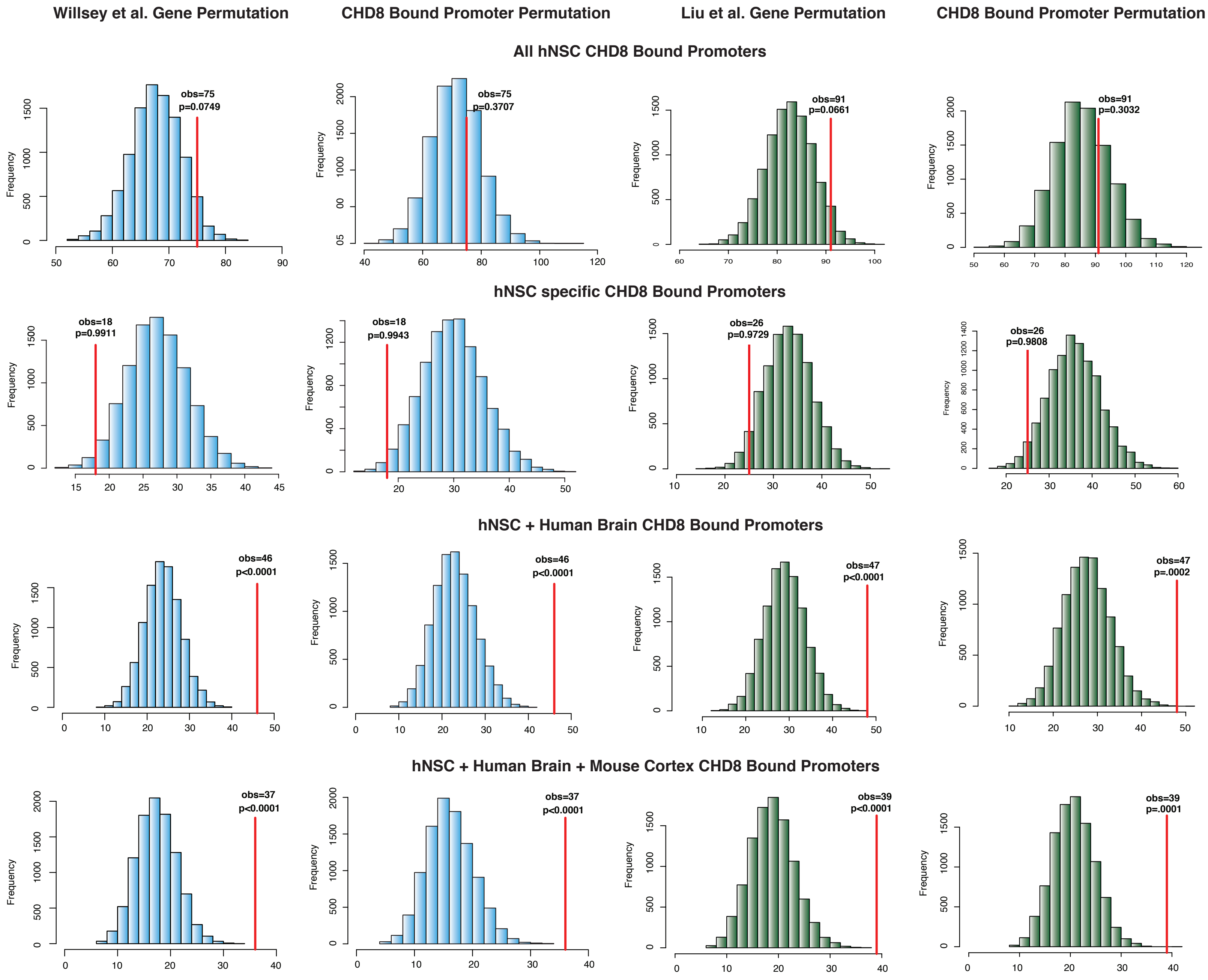

Supplementary Fig. 5. Enrichment analysis ASD risk genes in CHD8 targets. Analysis of Willsey et al. genes indicated by blue bars and Liu et al. genes by green bars. Left panels, Histograms of the number of ASD risk genes identified by CHD8 promoter binding in each indicated set after permuting ASD risk gene identities. Right panels, Histograms of ASD risk genes identified by CHD8 promoter binding in each indicated set after permuting CHD8 bound promoters (See Supplementary Table 3 for number of promoters in each set, n=10000 iterations). The vertical red line in each panel shows the actual observed number of ASD risk genes identified in each subset.

CHD8 promoter targets are enriched in midfetal PFC-MSC co-expression network during human neurodevelopment

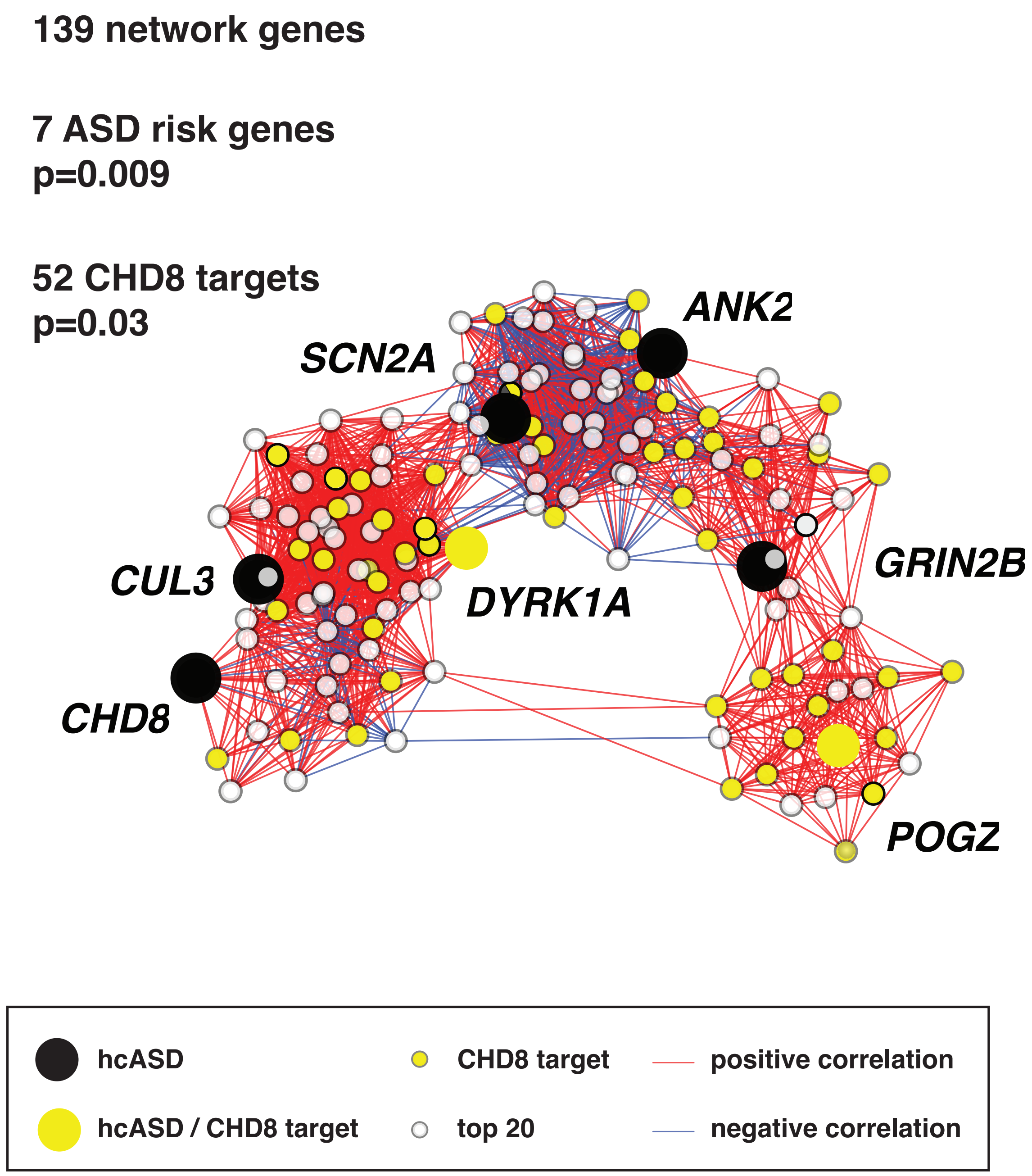

Supplementary Fig. 6. Human CHD8 targets are enriched in ASD-associated network. A gene co-expression network spanning 13-24 post conception weeks (defined as Periods 4-6 in Willsey et al.) was constructed as described (1,2), except the set of input genes was further restricted to only include genes exhibiting H3K27ac and/or H3K4me3 promoter marking in hNSCs to match the observed characteristics of CHD8 targets. The resulting network was tested for enrichment of potential ASD genes identified by Willsey et al., and genes with CHD8 binding sites in their promoters. The 20 genes best correlated with each high-confidence ASD gene ("hcASD gene") were included in the network provided the correlation value was R ≥ 0.7. The hcASD seed genes are shown as large circles; CHD8 targets are in yellow; and the top 20 genes that are not CHD8 targets are small white circles. The lines (edges) reflect co-expression correlations: positive correlations are in red and negative correlations are in blue.

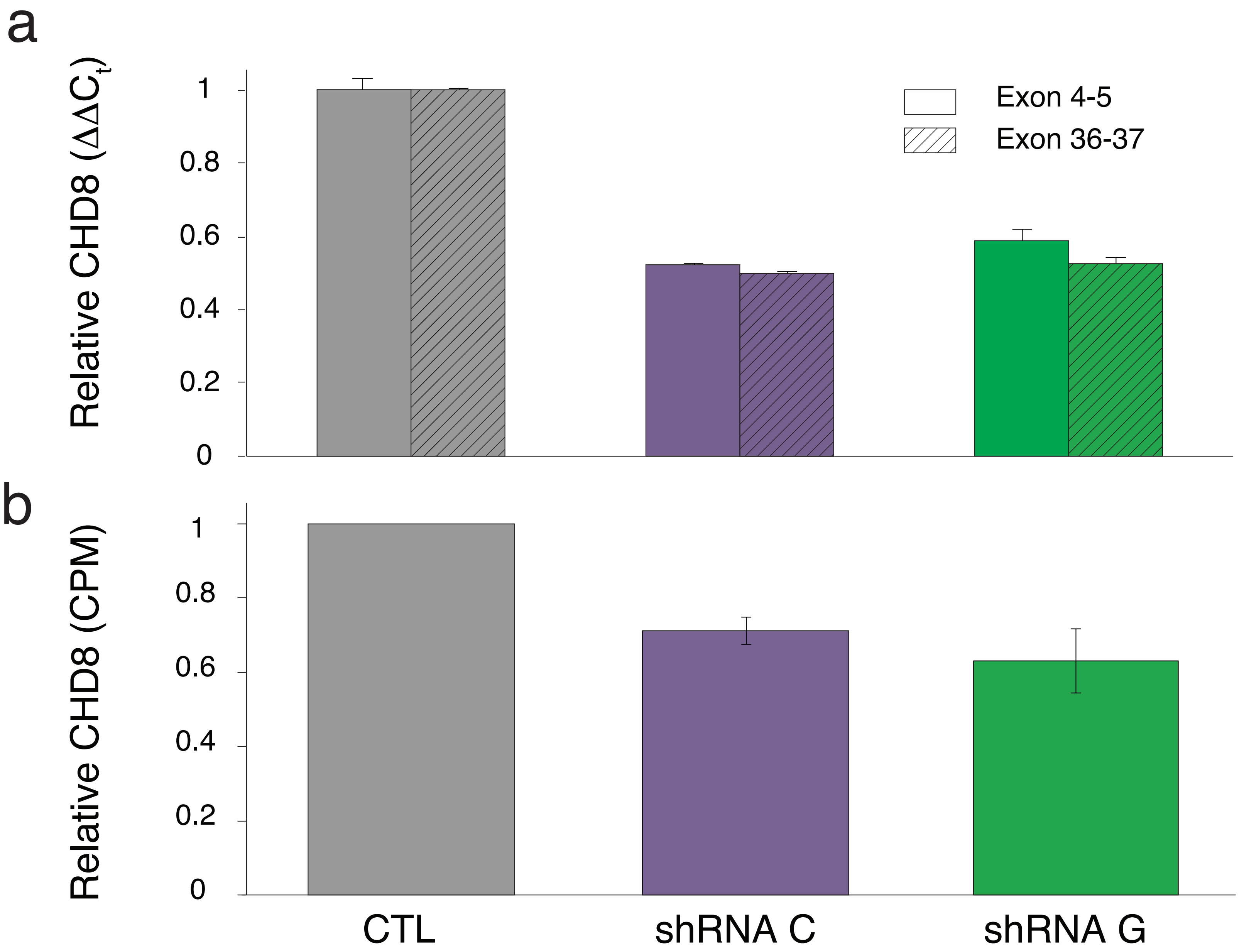

Supplementary Fig. 7. CHD8 transcript depletion by shRNA knockdown. a) QPCR relative quantification of CHD8 mRNA levels from hNSCs treated with control or CHD8 shRNA constructs. CHD8 mRNA levels were measured with two different primer sets targeting splice junctions of exons 4-5 or 36-37 of the CHD8 mRNA. Error bars represent standard deviation from three technical replicates of a single shRNA experiment. b) Relative count per million (CPM) quantification of CHD8 mRNA levels in knockdown RNA-seq experiments. Error bar represent standard deviation from four independent RNA-Seq experiments.

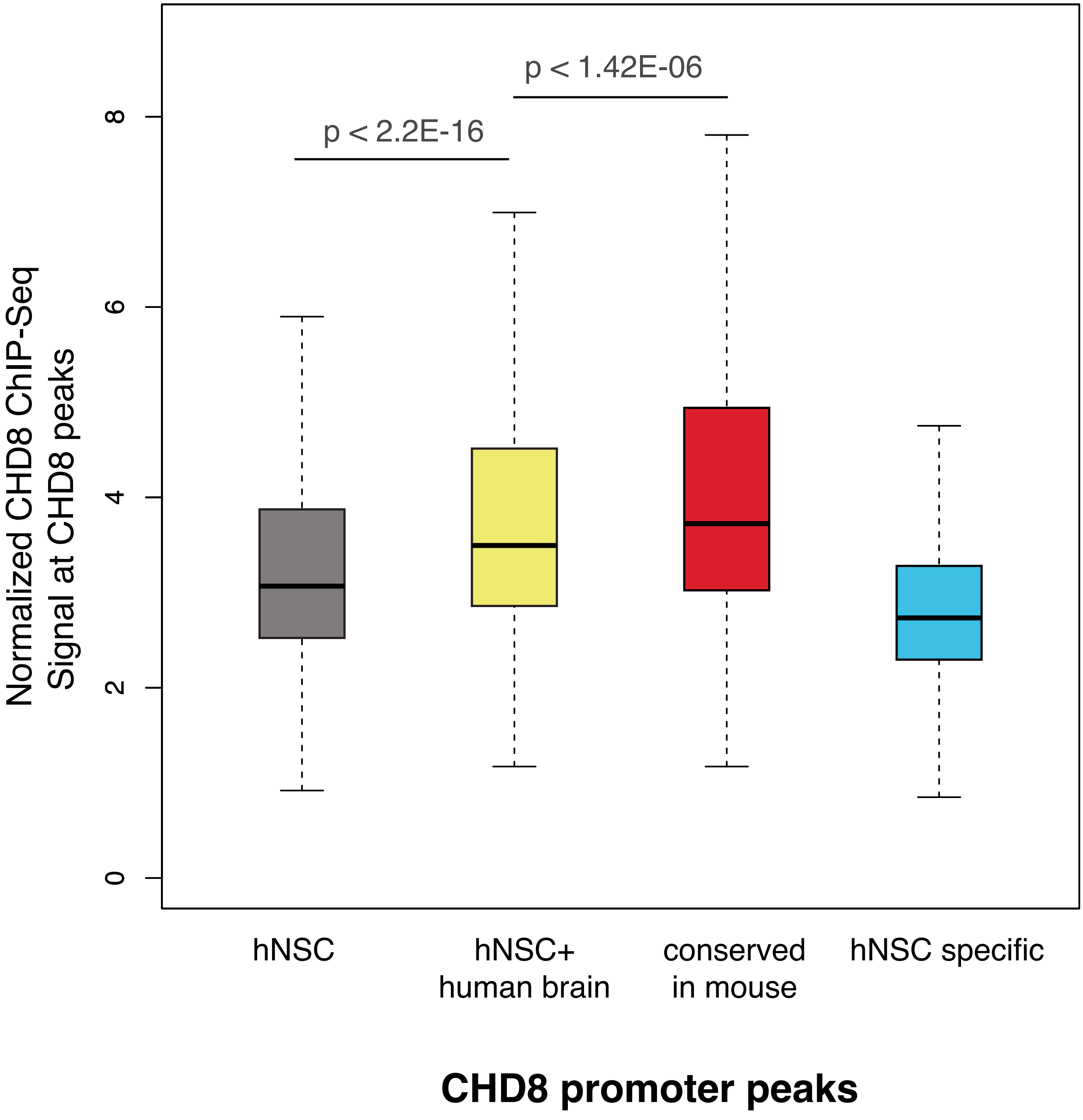

Supplementary Fig. 8. Conserved CHD8 targets have higher CHD8 ChIP-seq signal. Boxplot of the normalized CHD8 ChIP-seq signal for each subset of CHD8 target genes. Reported P values were calculated using Welch's t-test. Boxes represent middle 50% of values, while whiskers represent upper and lower 25% of values. Horizontal bar in each box represents median value.

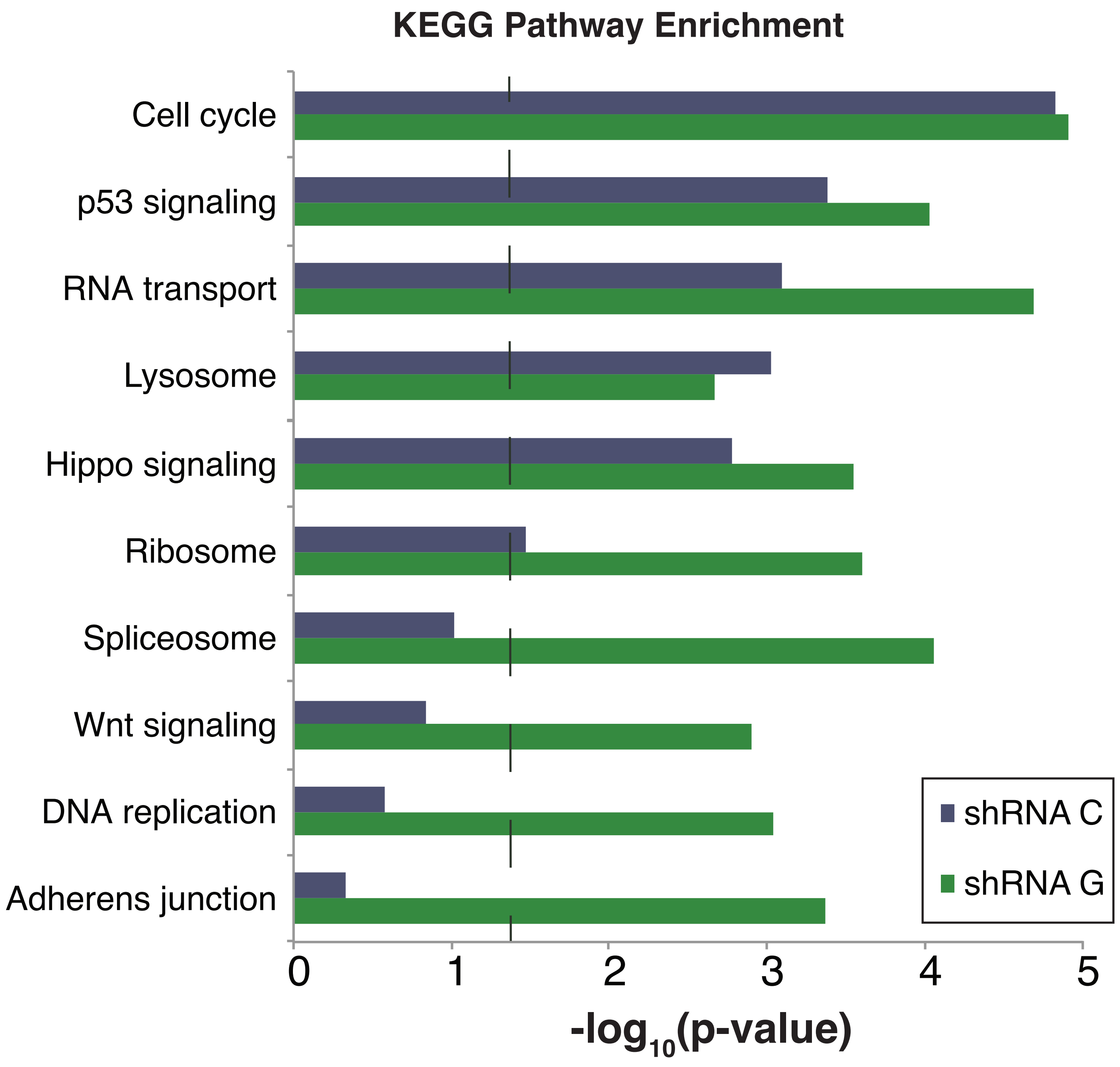

Supplementary Fig. 9. Biological pathways significantly affected by CHD8 knockdown. KEGG pathways showing significantly dysregulated gene expression for each shRNA knockdown. P values were calculated using Wilcoxon rank tests.

Hela Nuclear Lysate
Western with N and C
terminal CHD8 antibodies

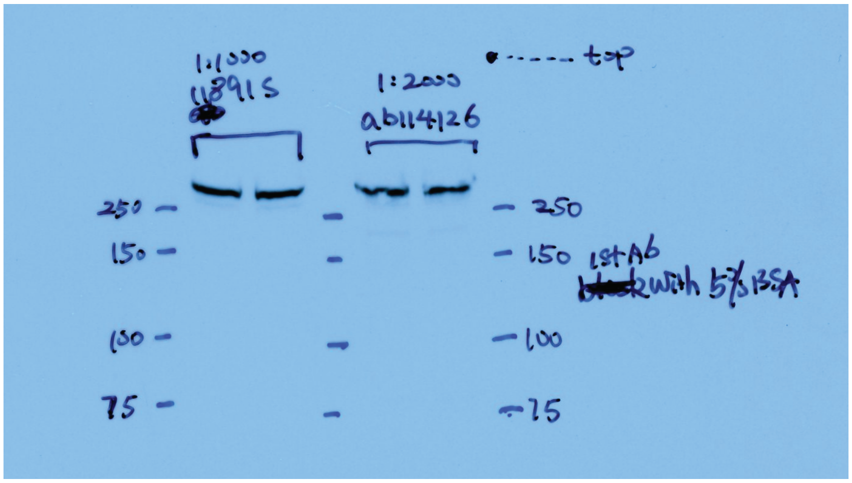

Hela IP-Western with N and C
terminal CHD8 antibodies
probed with CHD8-C antibody

(Image is flipped in main figure)

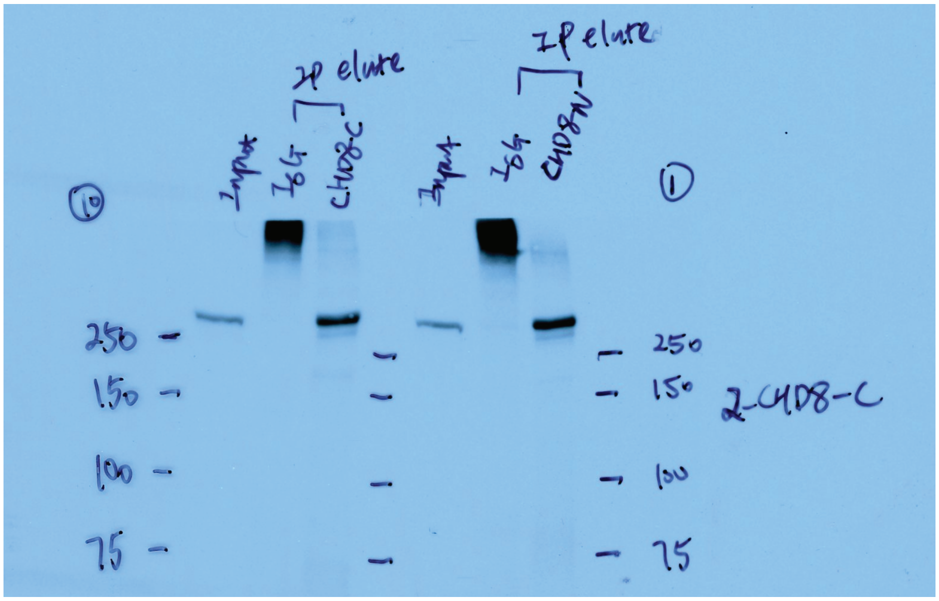

Western of hNSC whole cell lysates
with N terminal CHD8 antibodies
demonstrating CHD8 knockdown

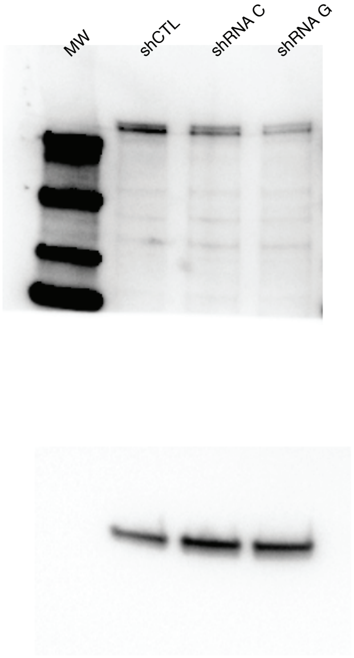

anti-CHD8-N

anti-actin

Supplementary Fig. 10. CHD8 western blots. Original CHD8 western blots supporting those shown in main and supplemental figures.

Supplementary References

1. Willsey, A.J. et al. Coexpression networks implicate human midfetal deep cortical projection neurons in the pathogenisis of autism. Cell 155, 997-1007 (2013).

2. Kang, H.J. et al. Spatio-temporal transcriptome of the human brain. Nature 478, 483-489 (2011).
